# A distinct holoenzyme organization for two-subunit pyruvate carboxylase

**DOI:** 10.1038/ncomms12713

**Published:** 2016-10-06

**Authors:** Philip H. Choi, Jeanyoung Jo, Yu-Cheng Lin, Min-Han Lin, Chi-Yuan Chou, Lars E. P. Dietrich, Liang Tong

**Affiliations:** 1 Department of Biological Sciences, Columbia University, New York, New York 10027, USA; 2 Department of Life Sciences and Institute of Genome Sciences, National Yang-Ming University, Taipei 112, Taiwan

## Abstract

Pyruvate carboxylase (PC) has important roles in metabolism and is crucial for virulence for some pathogenic bacteria. PC contains biotin carboxylase (BC), carboxyltransferase (CT) and biotin carboxyl carrier protein (BCCP) components. It is a single-chain enzyme in eukaryotes and most bacteria, and functions as a 500 kD homo-tetramer. In contrast, PC is a two-subunit enzyme in a collection of Gram-negative bacteria, with the α subunit containing the BC and the β subunit the CT and BCCP domains, and it is believed that the holoenzyme has α_4_β_4_ stoichiometry. We report here the crystal structures of a two-subunit PC from *Methylobacillus flagellatus*. Surprisingly, our structures reveal an α_2_β_4_ stoichiometry, and the overall architecture of the holoenzyme is strikingly different from that of the homo-tetrameric PCs. Biochemical and mutagenesis studies confirm the stoichiometry and other structural observations. Our functional studies in *Pseudomonas aeruginosa* show that its two-subunit PC is important for colony morphogenesis.

## Abstract (web summary)

Pyruvate carboxylases are homotetrameric enzymes in eukaryotes and most bacteria. Here, the authors report the structure of an unusual two-subunit form of the enzyme from the Gram-negative bacterium *Methylobacillus flagellates*, revealing an unexpected α_2_β_4_ stoichiometry.

## Graphical abstract

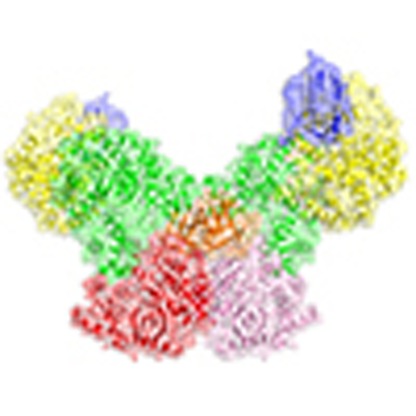

Pyruvate carboxylase (PC) catalyses the MgATP-dependent conversion of pyruvate to oxaloacetate^1^,^2^. It has important functions in gluconeogenesis, glyceroneogenesis, lipogenesis, neurotransmitter release, as well as anaplerosis to replenish the intermediates of the tricarboxylic acid (TCA) cycle. PC deficiency in humans is linked to various clinical symptoms such as lactic acidaemia and psychomotor retardation, while PC over-expression has been observed in several types of cancers^3^,^4^. The PC of the intracellular pathogen *Listeria monocytogenes* (LmPC) is important for growth and virulence in the host cell^5^,^6^.

PC is a member of the biotin-dependent carboxylase family and contains biotin carboxylase (BC), carboxyltransferase (CT) and biotin carboxyl carrier protein (BCCP) components. In mammals and most bacteria, these domains reside in a single polypeptide chain (Fig. 1a). These single-chain PC enzymes function as 500 kD homo-tetramers and are stimulated by acetyl-CoA. In mammals, insulin suppresses hepatic gluconeogenesis by reducing acetyl-CoA levels and thereby PC activity, suggesting an important role for PC in type 2 diabetes^7^. The crystal structures of several single-chain PCs have defined their overall holoenzyme architecture^6^,^8^,^9^. The four PC molecules are arranged in two layers, in the overall shape of a diamond (Fig. 1b). The two molecules in each layer of the structure have few interactions with each other. The holoenzyme is primarily formed through BC (Fig. 1c) and CT domain dimers between the layers, which are located at alternate corners of the diamond. The structures also reveal a new domain, named the PC tetramerization (PT) domain^9^ or allosteric domain^8^ that is important for tetramer formation by *Staphylococcus aureus* and human PCs (SaPC and HsPC) and for interaction with acetyl-CoA^8^,^9^,^10^. This domain is formed by two discontinuous segments of the protein—the linker between BC and CT and that between CT and BCCP (Fig. 1a).

On the other hand, in a diverse array of Gram-negative bacteria among the phyla Proteobacteria and Aquificae (Supplementary Fig. 1), the PC enzyme is encoded by two separate genes, with the BC domain residing in a 52 kD α subunit and the CT and BCCP domains in a 67 kD β subunit (Fig. 1a)^11^. The BC, CT and BCCP domains of these two-subunit PCs share high sequence identity with those of the single-chain PCs (∼50% for BC and ∼40% for CT and BCCP), but the sequence identity is <10% in the regions corresponding to the PT domain (Supplementary Figs 2 and 3). It is generally accepted that these two-subunit PCs form an α_4_β_4_ complex^12^, which would be equivalent to the homo-tetrameric single-chain PCs. In contrast to the single-chain PCs, however, the two-subunit enzymes are not stimulated by acetyl-CoA^13^.

The two-subunit PC from *Pseudomonas aeruginosa* (PaPC) is necessary for growth on C3 and C6 compounds^14^. In addition, transcriptional upregulation of PaPC and other genes by PycR, which is encoded immediately downstream of the PC operon in the genome, is important for maintaining *P. aeruginosa* lung infection in a rat model, and PycR has been proposed as a potential target for antimicrobial therapy^15^. The PC from *Pseudomonas fluorescens* is important for the expression of small RNAs that mediate secondary metabolism and the biosynthesis of antibiotic compounds^16^. The two-subunit PC from *Azotobacter vinelandii* is necessary for growth on minimal media with glucose or sucrose as the sole carbon source, and a deletion mutant accumulates the compound poly-β-hydroxybutyrate due to a slowdown of the TCA cycle^17^,^18^.

The crystal structure of the BC subunit of *Aquifex aeolicus* PC has been reported^19^, although currently there is no structure of the holoenzyme of a two-subunit PC. We report here the structures of the two-subunit PC from *Methylobacillus flagellatus* (MfPC). Surprisingly, our structures reveal an α_2_β_4_ stoichiometry for the holoenzyme, with one BC dimer and two CT dimers, and the overall architecture of the holoenzyme is strikingly different from that of the homo-tetrameric PCs. An α helix at the C terminus of the α subunit is surrounded by two four-stranded β-sheets from two β subunits, forming a domain that is remarkably similar to the BT (BC–CT interaction) domain found in propionyl-CoA carboxylase^20^, 3-methylcrotonyl-CoA carboxylase^21^ and acetyl-CoA carboxylase (ACC)^22^. Biochemical and mutagenesis studies confirm the stoichiometry, as well as other observations from the structure. Our functional studies in *P. aeruginosa* PA14 show that PC is important for colony morphogenesis and for growth on pyruvate and glucose as carbon sources.

## Results

### Structure determination

We screened through a collection of two-subunit PCs for their expression and crystallization behaviour, and produced crystals of MfPC^23^. MfPC has 65% amino-acid sequence identity with PaPC (Supplementary Figs 2 and 3). The α and β subunits were over-expressed in *Escherichia coli* using a bicistronic plasmid, with an N-terminal hexa-histidine tag on the α subunit. The two subunits readily formed a stable complex, which was then purified to homogeneity. Purified MfPC was confirmed to be fully biotinylated by streptavidin gel shift assay, and had robust PC enzymatic activity (Supplementary Fig. 4). We compared the catalytic activities of MfPC and PaPC with those of the single-chain PCs LmPC and SaPC. In contrast to the single-chain PCs and consistent with previous reports, MfPC and PaPC are not sensitive to activation by acetyl-CoA (Supplementary Fig. 4). However, MfPC and PaPC have ∼10-fold higher specific activity than LmPC and SaPC in the absence of acetyl-CoA. When acetyl-CoA is present, SaPC activity becomes comparable to those of the two-subunit PCs, while LmPC activity is still ∼5-fold lower.

The MfPC crystals diffracted only to 6.6 Å resolution even after extensive optimization. To improve the diffraction quality, we introduced three single-site mutations (K419A, E421A and E422A) in the β subunit designed to reduce the surface entropy^24^. These residues are located in a solvent-exposed region of the CT domain and are not strictly conserved among PC homologues (Supplementary Fig. 3). The mutations had no effect on the catalytic activity of the enzyme, but they only slightly improved the diffraction quality of the crystals.

We then removed the flexible B sub-domain (residues 131–201) of the BC domain and replaced it with a Gly–Ser–Ser–Gly linker. The B domain closes down over the BC active site during catalysis but is often disordered or has high temperature factors in PC crystal structures. This deletion mutant behaved identically to the wild-type enzyme during purification, and expectedly was inactive catalytically. A mutant protein having this deletion and the three single-site changes produced a different crystal form with better diffraction, allowing us to determine the crystal structure of the MfPC holoenzyme at 3.0 Å resolution (Supplementary Fig. 5) using the molecular replacement method with the SaPC structure^9^ as the search model. Several segments of the protein had poor electron density and were not modelled in the final structure, including the linker between the PT and BCCP domains and the linker between the CT and PT domains for one β dimer. Only three of the four BCCP domains were observed. The crystallographic statistics are summarized in Table 1.

Using the structure of this mutant as the search model, we also determined the structure of the MfPC with only the three single-site mutations (referred to hereafter as wild-type since the mutations did not affect the catalytic activity) at 6.6 Å resolution. The BC and CT domains were located individually with the molecular replacement method. After one round of refinement, there was electron density indicating the position of the PT domain, the B domain of BC and a BCCP domain in one of the CT active sites. Inclusion of these additional domains reduced the *R* factors of the model (Table 1). Because of the low-resolution nature of the data set, this structure was not refined further.

### Overall structure of two-subunit PC deletion mutant

**Figure 1:**
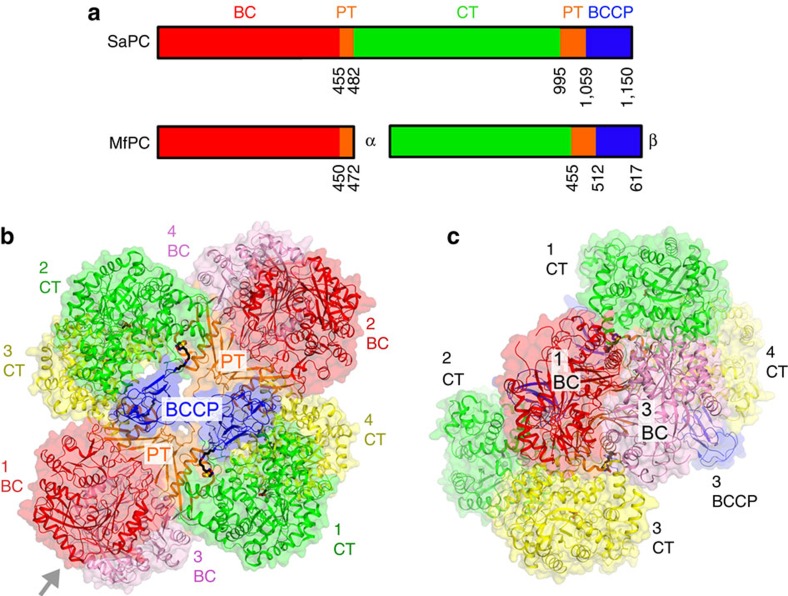
Domain organization of PC. (**a**) Domain organization of single-chain SaPC and two-subunit MfPC. The domains are labelled and given different colours. (**b**) Schematic drawing of the structure of the single-chain SaPC holoenzyme^9^. The domains in the four monomers are coloured according to **a** and labelled. (**c**) Structure of SaPC viewed down the two-fold axis of the BC dimer, along the arrow of **b**. The structure figures were produced with PyMOL (www.pymol.org).

**Figure 2:**
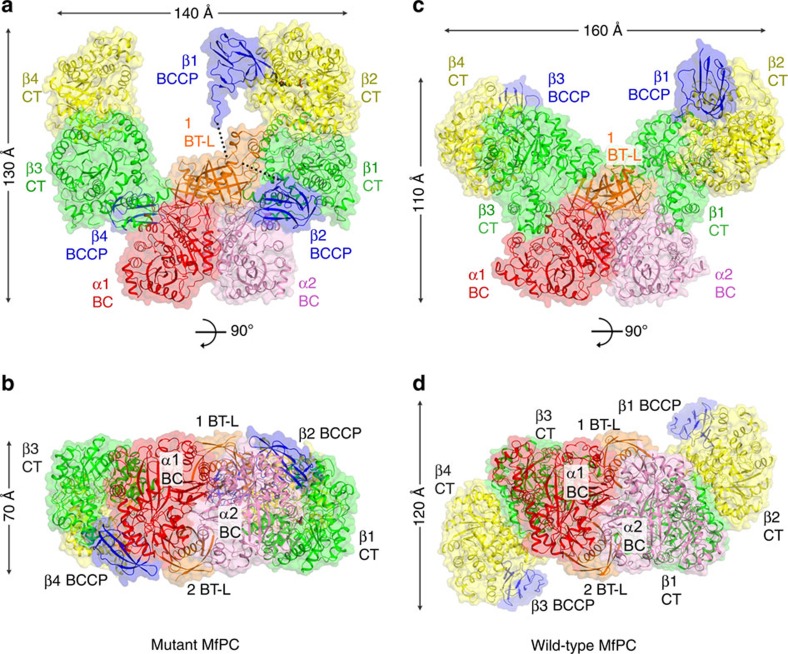
Structures of MfPC holoenzyme. (**a**) Schematic drawing of the mutant MfPC α_2_β_4_ holoenzyme structure, having the shape of the letter U in this view. The domains are coloured according to Fig. 1a, except the BC domain of the second α subunit is coloured in pink. BT-L, BT-like domain. (**b**) Structure of the mutant MfPC holoenzyme viewed down the BC domain dimer, along the arrow of **a**. (**c**) Structure of wild-type MfPC holoenzyme, having the shape of a butterfly in this view. (**d**) Structure of the wild-type MfPC holoenzyme viewed down the BC domain dimer, along the arrow of **c**. The two distal β subunits (β2 and β4) are splayed away from each other, and other differences to the mutant MfPC holoenzyme are also clearly visible by comparing with **b**.

Contrary to the expectations of an α_4_β_4_ holoenzyme, the structure revealed a 370 kD α_2_β_4_ oligomer for the MfPC mutant, with one BC dimer and two CT dimers (Fig. 2a). The mutant holoenzyme has overall dimensions of 140 × 130 × 70 Å and adopts a shape similar to the letter U, strikingly different from the holoenzyme architecture of single-chain PCs (Fig. 1b). The BC dimer (Fig. 2b) forms the bottom and a CT dimer is on each arm of the U shape, leaving a large cavity in the middle (Fig. 2a).

The BC dimer possesses a two-fold axis of symmetry, with root mean square (r.m.s.) distance of 0.1 Å for the equivalent Cα atoms of the two domains. However, the two CT dimers do not follow this symmetry, and have different orientation and position relative to the BC domains (Supplementary Fig. 6). This asymmetry would disfavour the binding of a second BC dimer at the top of the U-shaped structure, which partly explains the α_2_β_4_ stoichiometry observed in the structure. In addition, one of the CT dimers (β3 and β4) has relatively poor electron density and high temperature factors.

Of the three BCCP domains that are observed in this mutant holoenzyme, two are situated between the BC and CT domains, near the bottom of the U-shaped structure (Fig. 2a). These two BCCP domains likely come from the two β subunits (β2 and β4) that are distal from the BC dimer, and may help stabilize the overall structure of the mutant holoenzyme. The third BCCP domain is located in the active site of the distal β subunit with good electron density (β2), and its biotin is well ordered (Supplementary Fig. 5), but it comes from a proximal β subunit (likely β1). Therefore, the BCCP domains are extensively swapped in the holoenzyme.

We assigned the BCCP in the CT active site as coming from the other proximal β subunit based on considering the distances of the gaps. To provide evidence for this assignment, we separately purified wild-type α and β subunits, along with A49T and K581A mutant β subunits, which would inactivate the CT and BCCP domains, respectively (please see section below on structure-based mutations). The wild-type α subunit was then mixed separately with the three β subunits to form the holoenzyme. As expected, the wild-type holoenzyme was fully active, while the holoenzymes containing the A49T or K581A mutant β subunit had 1 and 2% of wild-type activity, respectively. We then diluted and mixed the two β subunit mutants together in a 1:1 ratio, allowing the formation of homo and hetero mutant β subunit dimers, before adding the α subunit to form a mixed population of hybrid mutant holoenzymes. If a BCCP domain can only visit the CT active site in the same β subunit during catalysis, we would expect no recovery of activity. However, if a BCCP domain visits the active site in the other β subunit during catalysis, about ∼25% of wild-type activity should be recovered. In support of the latter model and the assignments of BCCP in the structure, 29% of wild-type activity was recovered when the β subunit mutants were combined.

### Overall structure of wild-type two-subunit PC

The structure of the wild-type MfPC confirmed that the two-subunit PC holoenzyme has α_2_β_4_ stoichiometry (Fig. 2c). However, the CT dimers moved closer to the BC dimer and the two proximal β subunits moved close to each other as well, indicating that the contact between the BC and CT domains is flexible. Consequently, there is no space between the BC and CT domains to accommodate a BCCP domain and the large cavity in the centre of the mutant holoenzyme is absent as well. The wild-type holoenzyme instead has an overall shape of a butterfly, with overall dimensions of 160 × 110 × 120 Å (Fig. 2c). The two distal β subunits are splayed away from each other (Fig. 2d), which would further disfavour the binding of a second BC dimer.

Compared with the single-chain PCs, the CT dimers in MfPC (Fig. 2d) are rotated by almost 90° relative to those in SaPC (Fig. 1c and Supplementary Fig. 6). Therefore, the difference in the architecture of the two-subunit PC holoenzyme is not due simply to the removal of a BC dimer from one corner of the single-chain PC tetramer.

On the basis of the single-chain PC structures, we expected that the B domain of BC would not have interactions with other regions of the protein. However, the B domain in the wild-type MfPC structure has some contact with the proximal β subunit (Fig. 2c), due to the large differences in the orientation of the CT dimer compared with the single-chain PCs. The loss of this BC–CT interaction in the mutant protein may explain why the orientation of the CT dimers differs from that in the wild-type enzyme.

The structures of the individual BC and CT dimers of MfPC are largely similar to those of the single-chain PCs (Supplementary Fig. 7). The structure of the BC dimer is actually more similar to that of SaPC in complex with CoA^10^. The C-terminal helix is disordered in the structure of the BC subunit of *A. aeolicus* PC^19^.

### A BT-like structure in two-subunit PC

As in the single-chain PCs, there are few direct contacts between the BC and CT dimers in MfPC. Instead, interactions between the α and β subunits are mediated primarily through a helix at the C-terminal end of an α subunit (residues 454–472, outside of the BC domain) and a four-stranded anti-parallel β-sheet (residues 470–512) in the CT–BCCP linker of a β subunit, equivalent to the PT domain in the single-chain PCs (Fig. 1a), with r.m.s. distance of 3.7 Å for 56 equivalent Cα atoms between the MfPC and SaPC structures (Supplementary Fig. 7).

**Figure 3:**
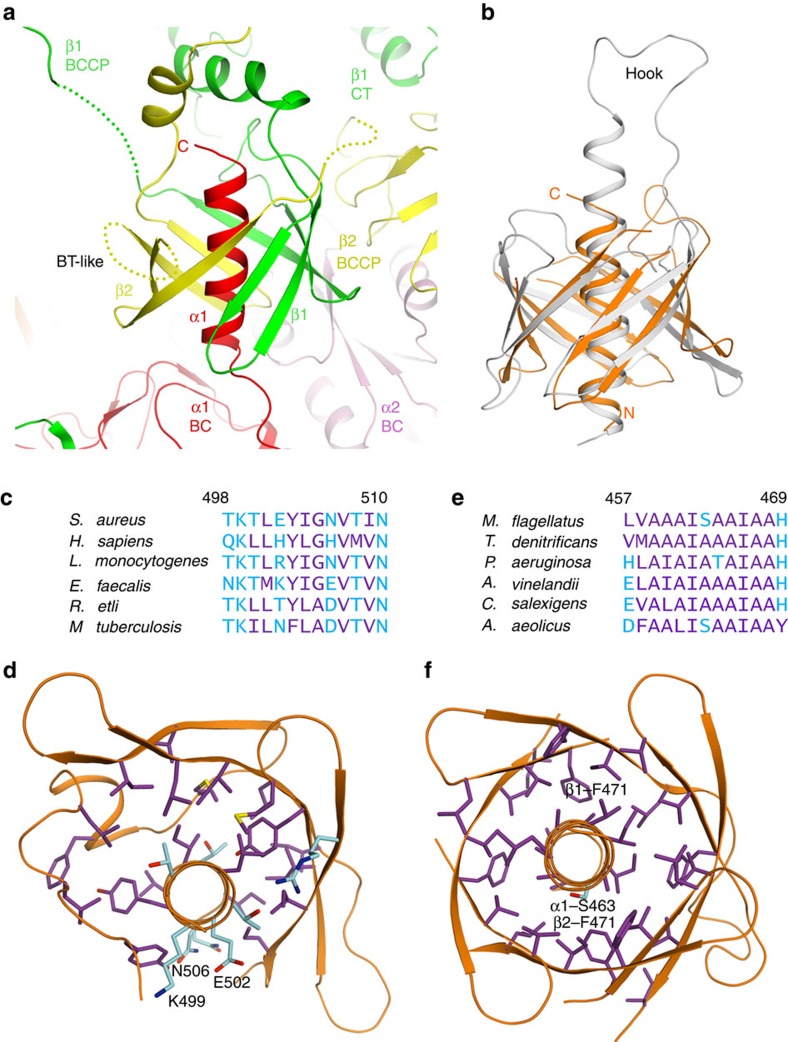
A BT-like domain in the structure of two-subunit PC. (**a**) Schematic drawing of the BT-like domain in MfPC. The helix comes from the α subunit (red), and the two four-stranded β-sheets come from two separate β subunits (green and yellow). Each subunit is given a different colour to highlight the origins of the protein segments in the BT-like domain. (**b**) Overlay of the structures of the BT-like domain of MfPC (orange) and the BT domain of PCC (grey)^20^. (**c**) Sequence alignment of the residues in the α helix of the PT domain in single-chain PCs. Hydrophobic residues are coloured purple, and hydrophilic ones in cyan. (**d**) The α helix of the PT domain in SaPC is amphipathic, with half of its surface having hydrophilic residues and exposed to the solvent. Hydrophobic side chains are coloured in purple, and hydrophilic ones in cyan. (**e**) Sequence alignment of the residues in the α helix of the BT-like domain in two-subunit PCs, at the C-terminal end of the α subunit. (**f**) The α helix of the BT-like domain in MfPC is hydrophobic, completely surrounded by the β-barrel and having mostly small side chains.

Surprisingly, the structure shows that the β-sheet from the second subunit of the CT dimer wraps around the other face of the helix (Fig. 2a), giving rise to a domain with a central α-helix surrounded by an eight-stranded up-down β-barrel (Fig. 3a and Supplementary Fig. 5). The overall structure of this domain is therefore remarkably similar to that of the BT (BC–CT interaction) domain found in the structures of propionyl-CoA carboxylase (PCC)^20^, 3-methylcrotonyl-CoA carboxylase^21^ and ACC^22^. The r.m.s. distance is 3.2 Å for 88 equivalent Cα atoms between MfPC and the PCC BT domain (Fig. 3b), even though the latter has a longer central helix and a ‘hook' linking the helix and the first strand of the barrel that is important for interactions in the PCC holoenzyme. The stoichiometry of this BT-like domain is α_1_β_2_, and the formation of this domain is likely the primary determinant for the α_2_β_4_ stoichiometry of the MfPC holoenzyme. While the BT-like domain has essentially no sequence identity with the PT domain of single-chain PCs, there is a high degree of sequence conservation of the BT-like domain among all bacterial two-subunit PCs (Supplementary Figs 2 and 3), strongly suggesting that these enzymes share a common α_2_β_4_ subunit composition.

An important difference between the two-subunit and single-chain PCs is that the helix in the PT domain of single-chain PCs is amphipathic, with the hydrophilic side exposed to the solvent in the holoenzyme (Fig. 3c,d). In contrast, the central helix of the BT-like domain in two-subunit PCs is entirely hydrophobic, consisting primarily of alanine residues (Fig. 3e), and is completely shielded from solvent by the β-barrel structure (Fig. 3f). This is similar to the BT domain in PCC and 3-methylcrotonyl-CoA carboxylase.

**Figure 4:**
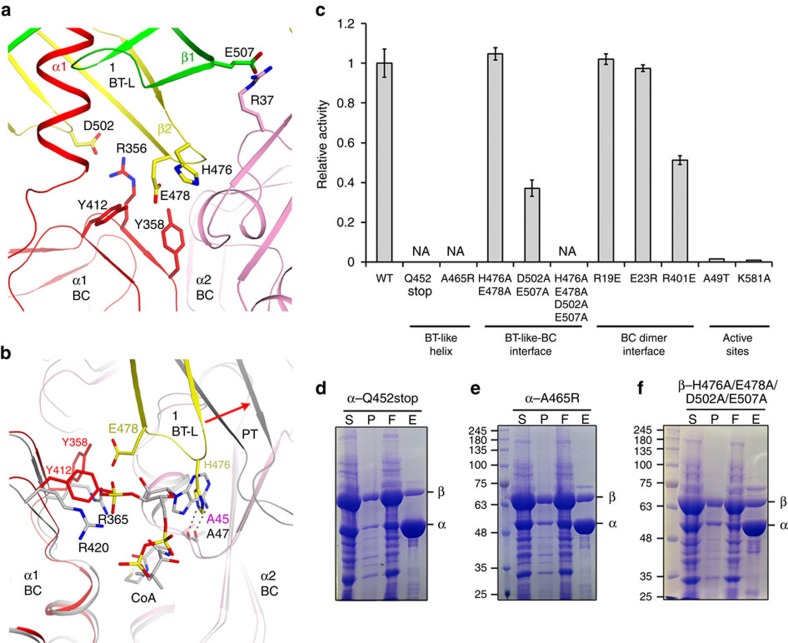
Mutagenesis studies to assess the structural observations. (**a**) Interactions between the BT-like domain and the BC dimer at the holoenzyme interface. (**b**) Overlay of the structure of MfPC (in colour) and SaPC (in grey) in complex with CoA (grey for carbon atoms)^10^. The red arrow indicates the difference in position of the BT-like domain in MfPC and the PT domain in SaPC. (**c**) Catalytic activities of wild-type and mutant MfPCs. The pyruvate concentration is at 20 mM. The error bars represent the s.d. from three independent measurements. NA, no activity observed under the condition tested. (**d**) Deletion of the α helix of the BT-like domain, or mutation of one of its Ala residues (A465R), abolished the formation of the MfPC holoenzyme. The two subunits were co-expressed in *E. coli*, with the α subunit carrying a His-tag. E, eluate from the Nickel column; F, flow-through of the Nickel column; P, pellet; S, soluble fraction. (**e**) Mutation of an Ala residue (A465R) in the helix of the BT-like domain abolished the formation of the MfPC holoenzyme. (**f**) A quadruple mutation in the interface between the BT-like domain and BC blocked holoenzyme formation.

A highly conserved 476-HGE-478 motif in a β-hairpin of the BT-like domain interacts with residues at the BC dimer interface (Fig. 4a). Additional conserved residues at this interface include Asp502 and Glu507 in the BT-like domain. However, these residues are not conserved in single-chain PCs (Supplementary Fig. 3). Instead, this region is equivalent to the binding site for the adenine diphosphate portion of acetyl-CoA in single-chain PCs (Fig. 4b)^8^,^9^,^10^. Especially, the side chain of His476 occupies the same position as the adenine base of acetyl-CoA, and forms an equivalent hydrogen bond with the carbonyl of Ala45 (Ala47 in SaPC). The Glu478 side chain is located near the 3′ phosphate of acetyl-CoA, and they both interact with residues in the opposite BC monomer. This is consistent with the fact that the BC dimer in MfPC is more similar to the CoA complex of SaPC (Supplementary Fig. 7), even though there is a significant difference in the position of the BT-like domain relative to the PT domain of single-chain PCs (Fig. 4b). This may also explain why two-subunit PCs are not sensitive to acetyl-CoA, as the binding site is occupied by a protein segment from the β subunit. The conformational change in the SaPC BC dimer induced by acetyl-CoA binding has also been linked to the activation of the enzyme^10^. Thus, the conformation of MfPC BC dimer induced by the 476-HGE-478 motif may be responsible for the high *k*_cat_ value observed for this enzyme (Supplementary Fig. 4).

### Further evidence for the α_2_β_4_ stoichiometry

The α_2_β_4_ stoichiometry of two-subunit PCs is unusual in light of the structures of the single-chain PCs along with the fact that all other biotin-dependent carboxylases studied to date have a 1:1 ratio between their BC and CT domains. Therefore, we sought further evidence in support of this stoichiometry.

**Figure 5:**
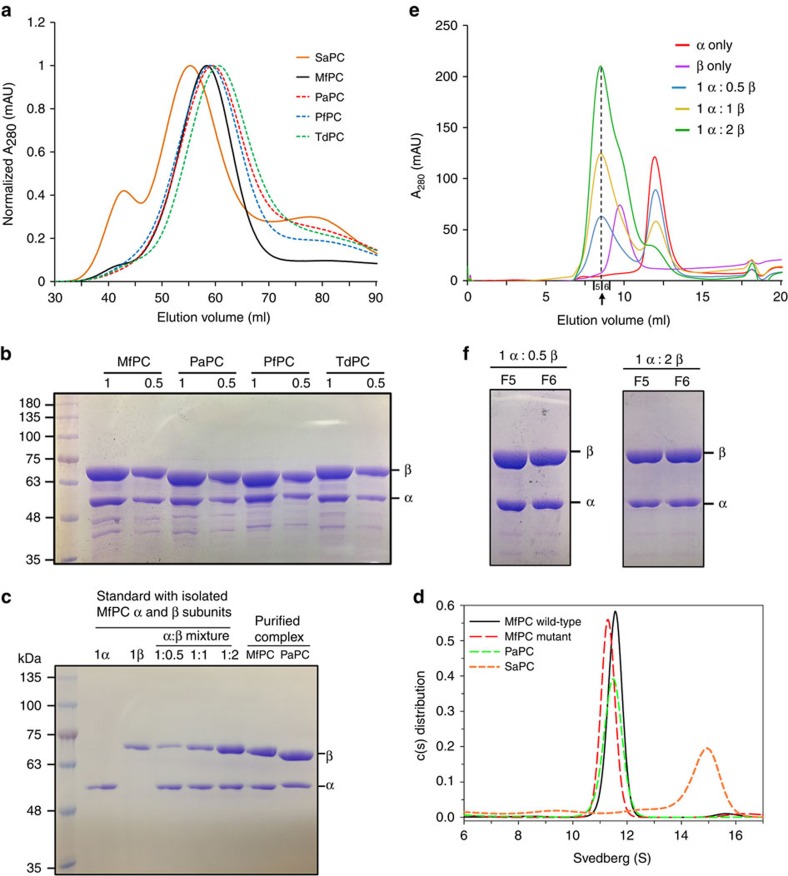
Experiments to confirm α_2_β_4_ stoichiometry of two-subunit PCs. (**a**) Gel filtration profiles for two-subunit PCs (Mf, *M. flagellates*; Pa, *P. aeruginosa*; Pf, *P. fluorescens*; Td, *T. denitrificans*) and the single-chain SaPC. (**b**) Coomassie-stained SDS–PAGE gel with purified two-subunit PCs from different species. Two lanes were run for each protein, with twice as much protein loaded in the first lane. The band for the α subunit in this lane is roughly the same as that for the β subunit in the second lane, consistent with the 1:2 stoichiometry. (**c**) SDS–PAGE gel with known amounts of α and β subunits and their mixtures, indicating that the purified holoenzyme has 1:2 stoichiometry. The β subunit in the MfPC holoenzyme sample is slightly smaller than the β subunit sample alone as it does contain a His tag. (**d**) Size distribution of PCs in solution at a concentration of 0.5 mg ml^−1^, based on the best-fit results by the continuous size distribution analysis. Two-subunit PCs (MfPC wild-type and deletion mutant, and PaPC) showed a similar major species of *S*=11.5, while single-chain SaPC showed a tetrameric species of *S*=14.8. (**e**) Gel filtration profiles from mixing experiments of different ratios of MfPC α and β subunits. The vertical dashed line indicates the elution volume of the complex that is formed on mixing. (**f**) Coomassie-stained SDS–PAGE gels of peak fractions collected from two different mixing experiments (F5 and F6 indicates collected fractions 5 and 6, respectively, indicated in **e**).

We confirmed that the α_2_β_4_ complex is not limited just to MfPC, as all the bacterial two-subunit PCs that we were able to express and purify from *E. coli* migrated at nearly the same position as MfPC on a gel filtration column, substantially later than that for the single-chain SaPC (Fig. 5a), and an SDS–polyacrylamide gel electrophoresis (SDS–PAGE) gel showed that the two-subunit PCs have the same ratio of α to β subunits (Fig. 5b). The 1:2 stoichiometry of the subunits is further confirmed by running known amounts of the individual subunits in an SDS–PAGE gel (Fig. 5c). To address the possibility that expression of the β subunit was somehow favoured over the α subunit in *E. coli*, we introduced a separate plasmid expressing only the α subunit along with the bicistronic plasmid expressing both α and β subunits. After purification, the complex was identical to that obtained from the bicistronic plasmid only, migrating at the same position on the gel filtration column and showing excess β by SDS–PAGE.

As further evidence in support of the α_2_β_4_ stoichiometry of two-subunit PCs, we carried out analytical ultracentrifugation experiments with MfPC (wild-type and the deletion mutant) and PaPC, and we used single-chain SaPC as a control. All three two-subunit PC samples gave molecular masses of ∼340 kD (∼11.5S), consistent with the α_2_β_4_ stoichiometry (Fig. 5d and Supplementary Fig. 8). In comparison, SaPC gave a molecular mass of ∼570 kD (14.8S), consistent with a homo-tetramer as we found earlier^25^.

We then purified the α and β subunits of MfPC separately and mixed them together in different molar ratios, followed by analytical gel filtration. Mixing of α and β in 1:2, 1:1 or 2:1 molar ratios did not change the composition of the complex as they all migrated similarly on the gel filtration column (Fig. 5e) and showed excess β over α by SDS–PAGE (Fig. 5f). Moreover, when α and β subunits were mixed in a 1:1 molar ratio, only half of the total α formed the complex and the rest migrated as free α on the gel filtration column. Only when α and β were mixed in a 1:2 molar ratio was all of the α subunit absorbed into the complex.

While it has been generally accepted that the bacterial two-subunit PCs form an α_4_β_4_ complex^12^, earlier biochemical characterizations of these enzymes from several species actually produced a range of estimates for the molecular weight and subunit composition^17^,^26^,^27^. Studies of the two-subunit PC from *A. vinelandii*, *Pseudomonas citronellolis* and *P. fluorescens* reported a molecular weight of ∼300 kD based on gel filtration and sedimentation experiments, and these groups proposed an α_2_β_2_ subunit stoichiometry^17^,^26^,^27^. On the other hand, another study on the PC enzyme from *P. citronellolis* reported a molecular weight of ∼500 kD based primarily on gel filtration analysis, and this group proposed an α_4_β_4_ subunit composition^12^. However, α_4_β_4_ subunit ratios of 1:1.7 and 1:1.18 were found based on two different protocols for Coomassie blue staining of SDS gels, which is inconclusive between 1:1 and 1:2 ratios of subunits. On the basis of SDS–PAGE gels, PaPC appears to have an excess of β subunit when it is expressed aerobically in *Pseudomonas*^11^. The wide range of these estimates is likely due to the limitations of using gel filtration to determine molecular weights, particularly for large proteins with extended shapes.

### BCCP movement during catalysis

The two-subunit PCs are unique among the biotin-dependent carboxylases in that they have a 1:2 ratio of BC:CT active sites. In both the wild-type and mutant structures, a BCCP domain is found only in the active site of the distal CT domain (Fig. 2a,c). This distal CT active site is located 70 Å away from the nearest BC active site (Supplementary Fig. 9), a distance which is within the range found in other biotin-dependent carboxylases, and which supports a swinging-domain model for catalysis^2^. The proximal CT domain active site is accessible to BCCP in the mutant structure, but inaccessible in the wild-type structure due to clashes with the BC dimer (Supplementary Fig. 9). This suggests the possibility that only the distal CT domain is catalytically active. In this model, one β subunit would provide the active CT domain, while the other β subunit provides the active BCCP domain.

We cannot rule out an alternative model in which conformational changes in the wild-type enzyme allow both CT domains to be involved in catalysis. The CT active site of the proximal β subunit is likely ∼40 Å away from the same BC active site that will be accessed by the distal β subunit (Supplementary Fig. 9), and therefore both BCCP domains of a β dimer likely visit the same BC active site during catalysis. It is not clear from the structures whether there would be coordination between the two BCCP domains in their access to this active site. It could be possible that while one BCCP domain is in the BC active site, the other BCCP domain is in the CT active site.

### Structure-based mutations block holoenzyme formation

We introduced mutations in MfPC based on the structural observations, and tested their effects on catalysis (Fig. 4c) and/or holoenzyme formation. As expected, deleting the last helix of the α subunit with the Q452stop mutant, in the centre of the BT-like domain, abolished the formation of the holoenzyme (Fig. 4d). In addition, mutating one of the alanine residues in this helix to arginine (A465R) also abrogated the formation of the holoenzyme, confirming that it is necessary for the helix to have small hydrophobic residues to form the intact BT-like domain (Fig. 4e). Both mutants are catalytically inactive as well (Fig. 4c). We then assessed the function of the conserved residues at the interface between the BC- and BT-like domains (Fig. 4a) by generating the double mutants H476A/E478A and D502A/E507A. Both of these double mutants still formed a stable holoenzyme, and the H476A/E478A mutant was fully active while the D502A/E507A double mutant retained 40% of wild-type activity (Fig. 4c). However, the H476A/E478A/D502A/E507A quadruple mutation completely abolished holoenzyme formation (Fig. 4f) and catalytic activity (Fig. 4c), consistent with the structural observations and implicating this interface as a key region for stabilizing the holoenzyme.

The BC dimer interface is similar to that observed in SaPC (Supplementary Fig. 7), as well as the BC subunit of *E. coli* ACC^28^,^29^. Mutational studies of SaPC demonstrated that a single point mutation (K442E) at the BC dimer interface is sufficient to disrupt tetramerization of the enzyme and abolish catalytic activity^25^. We introduced the equivalent mutation (R401E) in the α subunit of MfPC, but found that it still formed the α_2_β_4_ complex and had only a ∼50% reduction in catalytic activity compared with wild-type MfPC (Fig. 4c). We tested two additional BC dimer interface mutants, R19E and E23R. In SaPC, the R19E mutant forms a tetramer but does not have catalytic activity, while E23R formed aggregates. The R19E and E23R MfPC mutants were also able to form the holoenzyme and did not differ markedly from the wild-type enzyme in catalytic activity (Fig. 4c). Overall, these results indicate that the MfPC complex is more robust to mutations at the BC dimer interface compared with SaPC. The single-chain PC monomers are held together in the tetramer primarily by the BC and CT dimer interfaces (Fig. 1b). The additional inter-subunit interactions mediated by the BT-like domain in MfPC may allow for the complex to better tolerate these BC interface mutations and retain catalytic activity. The α subunit of MfPC alone is a monomer in solution (Fig. 5e), and the BC domain from SaPC alone is also a monomer and catalytically inactive^25^, indicating that additional interactions within the holoenzyme are necessary to form a stable BC dimer in these PC enzymes.

We also created the A49T mutant of MfPC, equivalent to the disease-causing A610T mutant of HsPC. This residue is located in the CT active site (Supplementary Fig. 9), and the bulkier Thr side chain is expected to interfere with biotin binding^10^. The A49T mutant had essentially no catalytic activity (Fig. 4c). In addition, blocking biotinylation of the enzyme by mutating the Lys581 residue in BCCP to Ala also abolished catalysis, as expected (Fig. 4c).

### Colony morphogenesis of *P. aeruginosa* PC mutant strains

**Figure 6:**
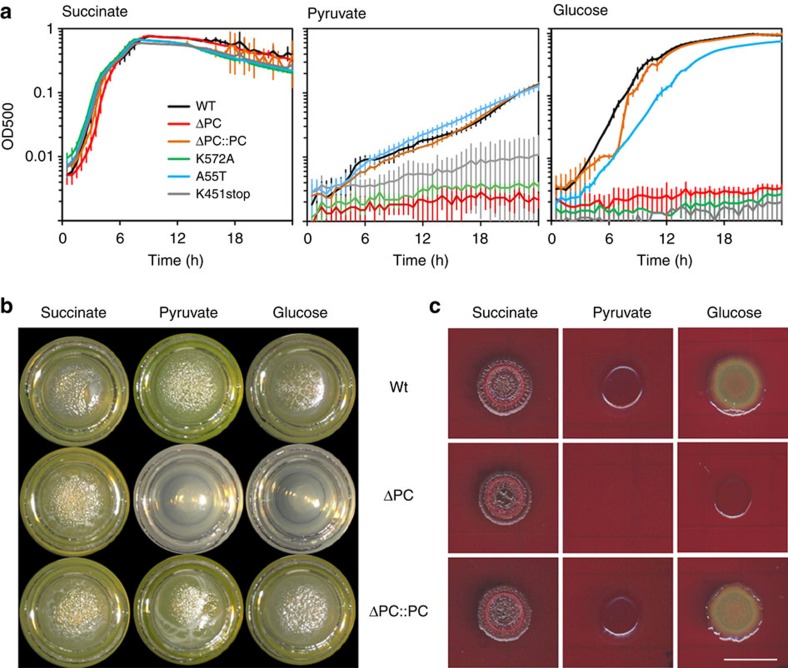
The two-subunit PaPC is required for growth on selected carbon sources. (**a**) Shaken liquid-culture growth of wild-type *P. aeruginosa* PA14, *ΔPC*, the PC-complemented strain and various site-specific mutants in a defined medium containing 20 mM succinate, pyruvate or glucose as the sole carbon source. Each growth curve represents the average of three biological replicates. Error bars denote s.d. (**b**) Pellicles formed by *P. aeruginosa* PA14, Δ*PC*, and the PC-complemented strain on a defined medium containing succinate, pyruvate, or glucose as the sole carbon source. (**c**) Colony morphology of *P. aeruginosa* PA14, Δ*PC* and the PC-complemented strain on a defined medium (supplemented with 1% agar, Congo red and Coomassie blue) containing succinate, pyruvate or glucose as the sole carbon source. Images depict day 3 of colony development.

We then assessed the functional importance of the two-subunit PC in the human pathogen *P. aeruginosa* (PaPC). PaPC has high sequence identity with MfPC (67% identity overall, 74% BC, 72% CT, 53% BT-like and 62% BCCP), and is likely to have the same α_2_β_4_ stoichiometry and architecture based on our gel filtration and sedimentation analysis (Fig. 5a,d). We created a markerless deletion of the PC operon, Δ*PC* (Δ*PA14_71720-PA14_71740*), and a series of point mutants that would allow us to assess the functional importance of key residues identified in the structure and that are conserved between *M. flagellatus* and *P. aeruginosa*. The growth profiles of these strains were analysed in a defined medium containing either succinate (a C4 compound), pyruvate (C3) or glucose (C6) as the sole carbon source. We did not observe any major effects when growth of the mutant strains was compared with that of the wild type on succinate (Fig. 6a). While all strains showed overall decreased growth on pyruvate, a subset of the strains, including Δ*PC*, exhibited severe growth defects relative to the wild type (Fig. 6b). The K451stop mutation in the α subunit (equivalent to Q452 in MfPC), which would delete the helix of the BT-like domain and disrupt holoenzyme formation, and the K572A mutation in the β subunit (equivalent to K581 in MfPC), which would abolish biotinylation, led to growth defects in pyruvate that were similar to that of the Δ*PC* deletion mutant. The A55T mutation in the β subunit (equivalent to A49 in MfPC), which reduces PC activity by 50-fold and would interfere with biotin binding to the CT active site, exhibited growth on pyruvate that was similar to the wild type. Finally, the growth defect of Δ*PC* was rescued when the PC operon was placed back into the endogenous locus (Fig. 6a). These phenotypes were recapitulated on glucose, though all strains showed better growth overall on this carbon source. Defective growth of mutants with impaired PC activity on C3 and C6 (but not C4) carbon sources confirms earlier findings^14^.

While culturing in well-aerated, homogenous liquid medium has been a traditional system for assessing microbial growth phenotypes, it does not represent the primary mode of growth outside the laboratory for most microbes. In non-laboratory habitats, microbes typically grow as communities of densely packed cells held together by a self-produced matrix. Cells in matrix-encased assemblages often exhibit unique metabolisms due to resource limitation. To examine a possible role for PC in *P. aeruginosa* growth under such conditions, we adapted defined media for use in two additional models that assess the production of extracellular matrix: (i) a pellicle assay, in which liquid cultures are incubated without shaking and biofilms develop at the air–liquid interface; and (ii) a colony morphology assay, in which cell suspensions are spotted on agar-solidified media containing the dyes Congo red and Coomassie blue and matrix production is required for the formation of wrinkle structures on the colony surface^30^,^31^. All strains formed robust pellicles and colony structures when succinate was supplied as the carbon source, with the Δ*PC* showing similar morphologies to those of the wild type. However, when glucose or pyruvate was provided as the carbon source, the Δ*PC* mutation prevented growth (Fig. 6b,c).

**Figure 7:**
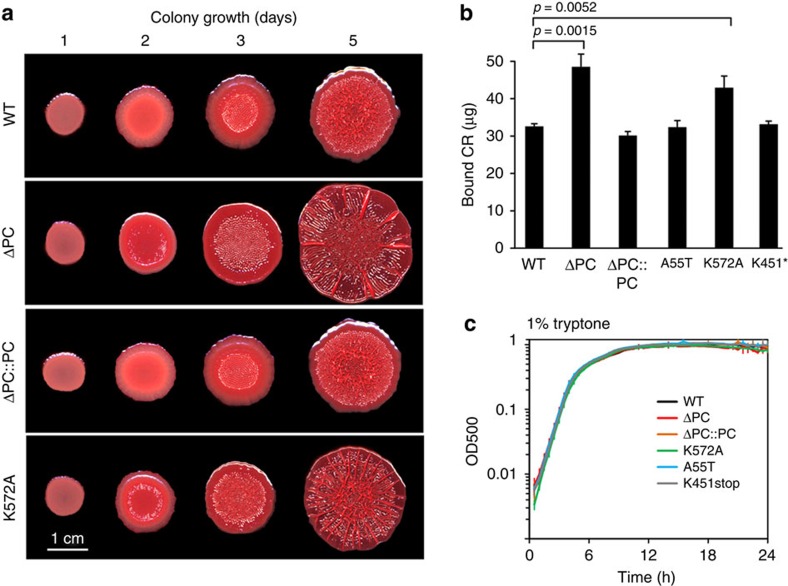
PaPC dysfunction leads to increased matrix production and altered colony morphology. (**a**) Colony morphology of wild-type *P. aeruginosa* PA14, *ΔPC*, the PC-complemented strain and the K572A mutant on 1% tryptone, 1% agar (supplemented with Congo red and Coomassie blue). (**b**) Quantification of Congo red binding, a proxy for matrix production, for *P. aeruginosa* PA14, *ΔPC*, the PC-complemented strain and various site-specific mutants grown on 1% tryptone, 1% agar with no added dyes. Colonies were grown for 3 days before they were collected for the Congo red binding assay. (**c**) Shaken liquid-culture growth of *P. aeruginosa* PA14, *ΔPC*, the PC-complemented strain and various site-specific mutants in 1% tryptone. Each growth curve represents the average of three biological replicates. Error bars denote s.d.

The growth phenotypes we observed in the pellicle and colony morphology assays recapitulated the results for growth in aerated liquid cultures. However, utilization of complex medium (1% tryptone) revealed a PC-dependent phenotype specific to matrix production in colonies (Fig. 7a,b) (since we did not observe robust pellicle formation for wild-type PA14 in standing cultures of 1% tryptone medium, we could not test for altered pellicle formation under these conditions). In the colony morphology assay, the wild type initially produces a smooth colony, which forms wrinkle structures in the centre starting on day 3 (Fig. 7a). By day 5, the wrinkles have spread over the colony, but a region of smooth morphology remains at the colony edge. We found that, in contrast, Δ*PC* and K572A colonies begin to wrinkle on day 2 and exhibit a flatter morphology with several exaggerated wrinkles in a ‘spoke' formation, which is reminiscent of morphologies that arise during electron acceptor-limited conditions^32^. Colonies formed by all other mutants exhibited morphologies that were similar to the wild type. Quantification of the cationic exopolysaccharide Pel, a major component of the PA14 biofilm matrix^33^, showed that, indeed, increased matrix production is associated with enhanced colony wrinkling (Fig. 7a,b). Wild-type colony morphology and matrix production were restored in the Δ*PC* complementation strain (Fig. 7a). Interestingly, colony morphology and liquid-culture growth phenotypes did not correlate. Though the Δ*PC* and K572A mutants showed altered colony morphogenesis on agar-solidified 1% tryptone, they were unaffected in shaken liquid-culture growth on this medium. Further, while both the K572A and K451stop mutants exhibit defects when grown on defined media containing pyruvate or glucose as the sole carbon source, only K572A shows altered development in the colony morphology assay on tryptone. The colony morphology assay therefore reveals PC-dependent metabolic disruptions that specifically affect a matrix-encased, structured community.

## Discussion

The central helix at the C-terminal end of the α subunit is not present in the BC subunit of bacterial multi-subunit ACC^34^. In fact, many of the PC α subunits are mis-annotated as BC subunits of ACC in the sequence database, and the presence of this C-terminal helix can be used to readily distinguish between them. In addition, the multi-subunit ACC and the two-subunit PC are both present in several bacterial species, including *P. aeruginosa*^2^, and this C-terminal helix may ensure that the correct α subunit is incorporated into the complex *in vivo*.

The PC enzymes from archaea are also separated into α and β subunits^35^,^36^, but they have several major differences from bacterial two-subunit PCs. The β subunit of archaeal PCs is ∼570 residues, significantly shorter than the 600–620 residues of bacterial two-subunit PCs. This is due to a large deletion in the region corresponding to the putative PT-/BT-like domain, such that only two β strands are predicted. The α subunit of bacterial two-subunit PCs is generally ∼471 residues and terminates precisely at the end of the BT-like domain α helix (Supplementary Fig. 2). In comparison, the α subunit of archaeal PCs has ∼500 residues due to a much longer C-terminal extension. In addition, while a stretch of small hydrophobic residues is present in the putative PT-/BT-like domain helix, it is much shorter than that in bacterial two-subunit PCs. Because of these significant differences, it is unclear if archaeal two-subunit PCs share the subunit stoichiometry and holoenzyme architecture of bacterial two-subunit PCs. Thus, it may be better to classify the bacterial and archaeal two-subunit PCs into two distinct categories.

The multifunctional biotin-dependent carboxylases likely arose through gene fusion of smaller mono-functional precursors. The structures of the homo-tetrameric and the two-subunit PCs give general insights into the mechanisms by which a protein can adapt to the gene fusion event, with the newly formed multi-domain protein preserving essential functions while gaining new features. An important consequence of this gene fusion event in the case of PC would be the disruption of the α_1_β_2_ stoichiometry of the BT-like domain, likely precipitating the drastic differences observed in holoenzyme architectures. However, the structures of the individual domains and the catalytic mechanism appear to be conserved between homo-tetrameric and two-subunit PCs. With the disruption of the BT-like domain, the new PT domain acquired additional functions, including tetramer stabilization and recognition of the allosteric activator acetyl-CoA. Intriguingly, acetyl-CoA binds to single-chain PCs in a conformation that mimics an important structural motif in the two-subunit PCs, suggesting that the development of acetyl-CoA regulation of PC might be linked to the structural rearrangements induced by the fusion. The various structures of biotin-dependent carboxylases have demonstrated that homologous domains can be arranged into remarkably different holoenzyme architectures, and that these distinct architectures have been tuned by evolutionary processes to perform distinct functions in cells.

Our *in vivo* results obtained with *P. aeruginosa* PA14 PC mutants confirm the role of PaPC during growth on pyruvate, a C3 compound, and support the model that this enzyme functions to replenish C4 intermediates thereby enabling TCA cycle function^14^. We also observed that impaired PC function led to increased matrix production and the development of flat colonies with pronounced wrinkle features on a medium containing tryptone as the carbon source. We have previously observed this phenotype under electron acceptor limitation^32^ and hypothesize that it is an adaptive community response to cellular redox imbalance. This suggests that the anaplerotic role of PC supports TCA cycle function and redox homeostasis during multicellular growth on complex carbon sources and may contribute to *P. aeruginosa* pathogenicity via this mechanism^15^.

## Methods

### Protein expression and purification

The α and β subunits for PC from several bacterial species, including *M. flagellatus*, *P. aeruginosa*, *P. fluorescens* and *Thiobacillus denitrificans* were amplified from genomic DNA (American Type Culture Collection) and sub-cloned into the pCDFduet vector (Novagen). The α subunit was sub-cloned into MCS1 with an N-terminal hexa-histidine tag, and the β subunit was sub-cloned into MCS2 with no tag. The internal B-domain deletion was made using overlapping PCR. Surface entropy reduction mutations were chosen based on the UCLA SerP server^24^. The individual α and β subunits for MfPC were sub-cloned into pET28a with an N-terminal hexa-histidine tag. All expression constructs were co-transformed into BL21 Star (DE3) cells along with a plasmid encoding the *E. coli* biotin ligase (BirA) gene.

The cells were cultured in Luria–Bertani (LB) medium with 50 mg ml^–1^ streptomycin and 35 mg ml^–1^ chloramphenicol, and were induced for 14 h with 1 mM isopropyl-β-D-thiogalactosideat 20 °C. Before induction, 20 mg l^–1^ biotin and 10 mM MnCl_2_ were added to the growth medium. The protein was purified through nickel-agarose affinity chromatography (Qiagen) followed by gel filtration chromatography (Sephacryl S-300, GE Healthcare). The purified protein was concentrated to 15 mg ml^–1^ in a buffer containing 20 mM Tris (pH 8.0), 150 mM NaCl, 5% (v/v) glycerol and 5 mM dithiothreitol, flash-frozen in liquid nitrogen and stored at –80 °C. The protein was confirmed to be fully biotinylated by a streptavidin gel-shift assay. The N-terminal hexa-histidine tag was not removed for crystallization.

### Protein crystallization

Crystals were grown by the sitting-drop vapour diffusion method at 20 °C. For MfPC with B-domain deletion, the protein was incubated with 2.5 mM pyruvate for 30 min at 4 °C before crystallization set-up. The reservoir solution contained 19% (w/v) PEG3350, 2% tacsimate (pH 6.0) (Hampton) and 3% (v/v) ethanol. The crystals appeared within 1 week and grew to full size after an additional week. The crystals were cryo-protected in the reservoir solution supplemented with 10% (v/v) ethylene glycol and 5% (w/v) sucrose and were flash-frozen in liquid nitrogen for data collection at 100 K.

For wild-type MfPC, the protein at 15 mg ml^–1^ was incubated with 2.5 mM ATP and 2.5 mM pyruvate for 30 min at 4 °C before crystallization set-up. The reservoir solution contained 1.3 M ammonium sulfate and 0.1 M sodium citrate (pH 6.0). The crystals appeared after 1 day and grew to full size within a week. They were cryo-protected in the reservoir solution supplemented with 15% (v/v) ethylene glycol and flash-frozen in liquid nitrogen for data collection at 100 K

### Data collection and structure determination

X-ray diffraction data were collected at the Advanced Photon Source beamline NE-CAT 24-ID-E using an ADSC Q315r detector and at the X25 beamline at the National Synchrotron Light Source at Brookhaven National Laboratory using a Pilatus 6M detector. The diffraction images were processed using HKL2000 (ref. 37).

Crystals of the mutant MfPC belong to space group *R*32 with unit cell parameters of *a*=*b*=285.8 Å and *c*=274.9 Å. With an α_2_β_4_ oligomer in the asymmetric unit, the *V*_m_ is 2.9 Å^3^ Da^–1^ and the solvent content is 58%. The structure was solved by the molecular replacement method with the programme Phaser^38^, using the BC, CT and BCCP domains of the *S. aureus* PC structure^9^ as the search models. Manual rebuilding was carried out with Coot^39^ and refinement with the programme Refmac^40^.

Crystals of wild-type MfPC belong to space group *P*3_1_21 with unit cell parameters of *a*=*b*=160.8 Å and *c*=227.7 Å. A molecular replacement solution for this structure was found with the programme Phaser using the individual BC and CT domains of the refined mutant MfPC structure as the search models. There is one α subunit and two β subunits in the asymmetric unit, and the full α_2_β_4_ complex can be generated by a crystallographic two-fold axis. On the basis of this molecular replacement solution, the solvent content of the crystal is 73% and the Matthews coefficient is 4.6 Å^3^ Da^–1^. After one round of refinement, electron density was observed indicating the positions of the B domain of BC and the BT-like domain. There was also density indicating a BCCP domain in the active site of the distal CT domain, which itself adopts a conformation that is consistent with an interaction with BCCP. In addition, the MfPC molecules in the crystal do not have direct contacts without this BCCP domain, further supporting its placement in the model. After inclusion of these additional domains, we observed a decrease in the *R* values and clear density for these domains on refinement.

### Analytical ultracentrifugation

Analytical ultracentrifugation was performed on an XL-A analytical ultracentrifuge (Beckman Coulter) using an An-50 Ti rotor. The sedimentation velocity experiments were carried out using a double-sector *epon* charcoal-filled centrepiece at 20 °C with a rotor speed of 42,000 r.p.m. Protein solutions of 0.5 mg ml^–1^ (330 μl) and reference (370 μl) solutions, both containing 20 mM Tris (pH 8.0) and 150 mM NaCl, were loaded into the centrepiece. The absorbance at 280 nm was monitored in a continuous mode with a time interval of 360 s and a step size of 0.003 cm. Multiple scans at different time intervals were then fitted to a continuous c(s) distribution model using the SEDFIT programme^41^.

### Mutagenesis and kinetic studies

Mutants were made using the QuikChange kit (Stratagene) and confirmed by sequencing. They were expressed and purified using the same protocol as described for the wild-type enzyme. The catalytic activity was determined based on a published protocol^42^, which couples oxaloacetate production to the oxidation of NADH by malate dehydrogenase, followed spectrophotometrically by the decrease in absorbance at 340 nm. The activity was measured at room temperature in a reaction mixture containing 20 mM Tris (pH 7.5), 200 mM NaCl, 5 mM MgCl_2_, 50 mM sodium bicarbonate, 50 mM ammonium sulfate, 5 units of malate dehydrogenase (Sigma), 2 mM ATP, 108 nM MfPC (based on the α subunit) and varying concentrations of pyruvate. The Michaelis–Menten curve was fitted using the programme Origin.

### Construction of PC mutant and complementation strains

PC mutant and complementation strains were made for the two-gene operon encoding PC (*PA14_71720-PA14_71740*) in *P. aeruginosa* PA14 as follows. Relevant genomic sequences were amplified, with point mutations introduced where noted in Supplementary Table 1, and recombined into the allelic replacement vector pMQ30 through gap repair cloning in the *Saccharomyces cerevisiae* strain InvSc1. The deletion construct contained two fused ∼1-kb sequences representing regions upstream and downstream of the *PA14_71720-PA14_71740* operon. Point-mutant and complementation constructs contained the full operon sequence, with mutations where appropriate, plus these two flanking regions. Each plasmid was transformed into *E. coli* strain DH5α, verified by sequencing, and put into *P. aeruginosa* using biparental conjugation. PA14 single recombinants were selected on LB plates containing 100 μg ml^–1^ gentamicin. Double recombinants (with the final genotype of interest) were selected on agar plates containing 10% (w/v) sucrose, and their genotypes were confirmed by PCR. For point mutant strains, genotypes were confirmed by sequencing.

### *P. aeruginosa* PA14 growth conditions

For genetic manipulation and pre-culturing, PA14 was routinely grown in LB (unless otherwise noted) at 37 °C with shaking at 250 r.p.m. For growth curve analysis, overnight pre-cultures of PA14 strains were diluted 100-fold in either 1% tryptone or a defined medium (50 mM MOPS (4-morpholinepropanesulfonic acid), 43 mM NaCl, 93.5 mM NH_4_Cl, 2.2 mM KH_2_PO_4_, 1 mM MgSO_4_ and 1 μg ml^–1^ FeSO_4_) amended with 20 mM sodium succinate, sodium pyruvate or glucose as the carbon source and grown to the following approximate OD_500nm_ values: 0.6 for succinate; 0.1 for pyruvate; and 0.1 for glucose. These cultures were diluted to OD_500nm_≈0.01 and dispensed into 96-well plates, then incubated at 37 °C with continuous shaking on the medium setting in a Biotek Synergy 4 plate reader.

### *P. aeruginosa* PA14 colony morphology assay

For the standard assay, PA14 was grown overnight in LB and diluted to OD_500nm_=0.5. A volume of 10 μl of these normalized cell suspensions were spotted on colony morphology assay medium (1% tryptone, 1% agar, 40 μg ml^–1^ Congo red and 20 μg ml^–1^ Coomassie blue; 60 ml in each 9 cm × 9 cm square plate) and incubated in a humidified chamber at 25 °C for up to 5 days. When defined media were used for the colony morphology assay, PA14 pre-cultures were grown overnight in MOPS liquid medium (50 mM MOPS, 43 mM NaCl, 93.5 mM NH_4_Cl, 2.2 mM KH_2_PO_4_, 1 mM MgSO_4_ and 1 μg ml^–1^ FeSO_4_ with 20 mM succinate). Pre-cultures were centrifuged for 1 min at 12,396 × g and resuspended in defined medium without a carbon source to OD500_nm_=0.25. A volume of 10 μl of washed and resuspended cells were spotted onto solidified defined medium (25 mM HEPES (4-(2-hydroxyethyl)-1-piperazineethanesulfonic acid), 7.6 mM (NH_4_)_2_SO_4_, 0.8 mM MgSO_4_·7H_2_O, 10 mM K_2_HPO_4_, 20 mM of carbon source (succinate, pyruvate or glucose), 40 μg ml^–1^ Congo red, 20 μg ml^–1^ Coomassie blue and 1% agar; 60 ml in each 9 cm × 9 cm square plate) and incubated in a humidified chamber at 25 °C for up to 5 days. Images were taken with a CanoScan 5600F scanner (Canon).

### *P. aeruginosa* PA14 Congo red binding assay

PA14 colonies were grown for 3 days (76 h) in the standard colony morphology assay, with the modification that dyes were omitted from the 1% tryptone, 1% agar medium. Each colony was scraped from the agar using a 1,000 μl pipette tip and resuspended in 1.5 ml phosphate-buffered saline (136 mM NaCl, 2.68 mM KCl, 10.1 mM Na_2_HPO_4_ and 1.76 mM KH_2_PO_4_, at pH 7.4) supplemented with 60 μg of Congo red. Each colony suspension was briefly vortexed and shaken at 250 r.p.m. at 37 °C for 1 h to allow the matrix to bind the Congo red dye. The colony biomass was then pelleted by centrifugation at 16,873 × g for 2 min. A volume of 200 μl of supernatant were dispensed into 96-well plates and the absorbance at 490 nm, representing unbound Congo red, was measured using a Biotek Synergy 4 plate reader. Bound Congo red was calculated by subtracting the absorbance of the unbound Congo red from that of the control solution containing 60 μg Congo red per 1.5 ml PBS.

### *P. aeruginosa* PA14 pellicle assay

Overnight LB pre-cultures of PA14 strains were diluted 100-fold in MOPS liquid medium (50 mM MOPS, 43 mM NaCl, 93.5 mM NH_4_Cl, 2.2 mM KH_2_PO_4_, 1 mM MgSO_4_ and 1 μg ml^–1^ FeSO_4_) amended with 20 mM sodium succinate as the carbon source and grown to OD_500nm_≈0.7 A sample (4.6 ml) of each subculture was centrifuged at 10,000 × g for 1 min, and the pellet was resuspended in 23 ml of the defined medium amended with 20 mM sodium succinate, D-glucose or sodium pyruvate in a scintillation vial, with a starting OD_500nm_ at 0.14 for pellicle growth. Scintillation vials were incubated without shaking or disturbance at 37 °C for up to 4 days and photographed under side illumination using an iPhone 5S.

### Data availability

Atomic coordinates and structure factors for the two reported structures of *M. flagellatus* PC have been deposited in the Protein Data Bank under the primary accession code 5KS8. The authors declare that all other relevant data supporting the findings of this study are available on request.

## Additional information

**How to cite this article:** Choi, P. H. *et al*. A distinct holoenzyme organization for two-subunit pyruvate carboxylase. *Nat. Commun.* 7:12713 doi: 10.1038/ncomms12713 (2016).

## Supplementary Material

Supplementary Information Supplementary Figures 1-9 and Supplementary Table 1

## Figures and Tables

**Table 1:** Data collection and refinement statistics.

	**MfPC (B domain of BC deletion, K419A, E421A and E422A mutation)**	**MfPC (K419A, E421A and E422A mutation)**
*Data collection*		
Space group	*R*32	*P*3_1_21
Cell dimensions		
*a*, *b*, *c* (Å)	285.8, 285.8, 274.9	160.8, 160.8, 227.7
*α*, *β*, *γ* (°)	90, 90, 120	90, 90, 120
Resolution (Å)	50–3.0 (3.1–3.0)*	50–6.6 (6.83–6.6)
*R*_merge_	8.2 (60.8)	8.8 (>100)
CC_1/2_	(0.67)	(0.64)
*I*/*σI*	11.0 (1.8)	14.9 (1.3)
Completeness (%)	96 (96)	95 (96)
Redundancy	2.8 (2.7)	6.4 (6.3)
*Refinement*		
Resolution (Å)	50–3.0	50–6.6
No. reflections	77,247	6,031
*R*_work_/*R*_free_	22.5/27.2	27.4/33.4
No. of atoms		
Protein	21,658	11,277
Ligand/ion	30	0
Water	0	0
*B*-factors		
Protein	111	328
Ligand/ion	77	—
Water	—	—
R.m.s deviations		
Bond lengths (Å)	0.010	0.010
Bond angles (°)	1.5	1.3

Three crystals were used for data collection for mutant MfPC, and one crystal for wild-type MfPC.

^*^Highest-resolution shell is shown in parenthesis.
