# Supplementary material for: A distinct holoenzyme organization for two-subunit pyruvate carboxylase
Source: Nat Commun. 2016 Oct 6;7:12713. doi: 10.1038/ncomms12713 (PMC5059739; doi:10.1038/ncomms12713)
Supplement: Supplementary Information — Supplementary Figures 1-9 and Supplementary Table 1 [file ncomms12713-s1.pdf]

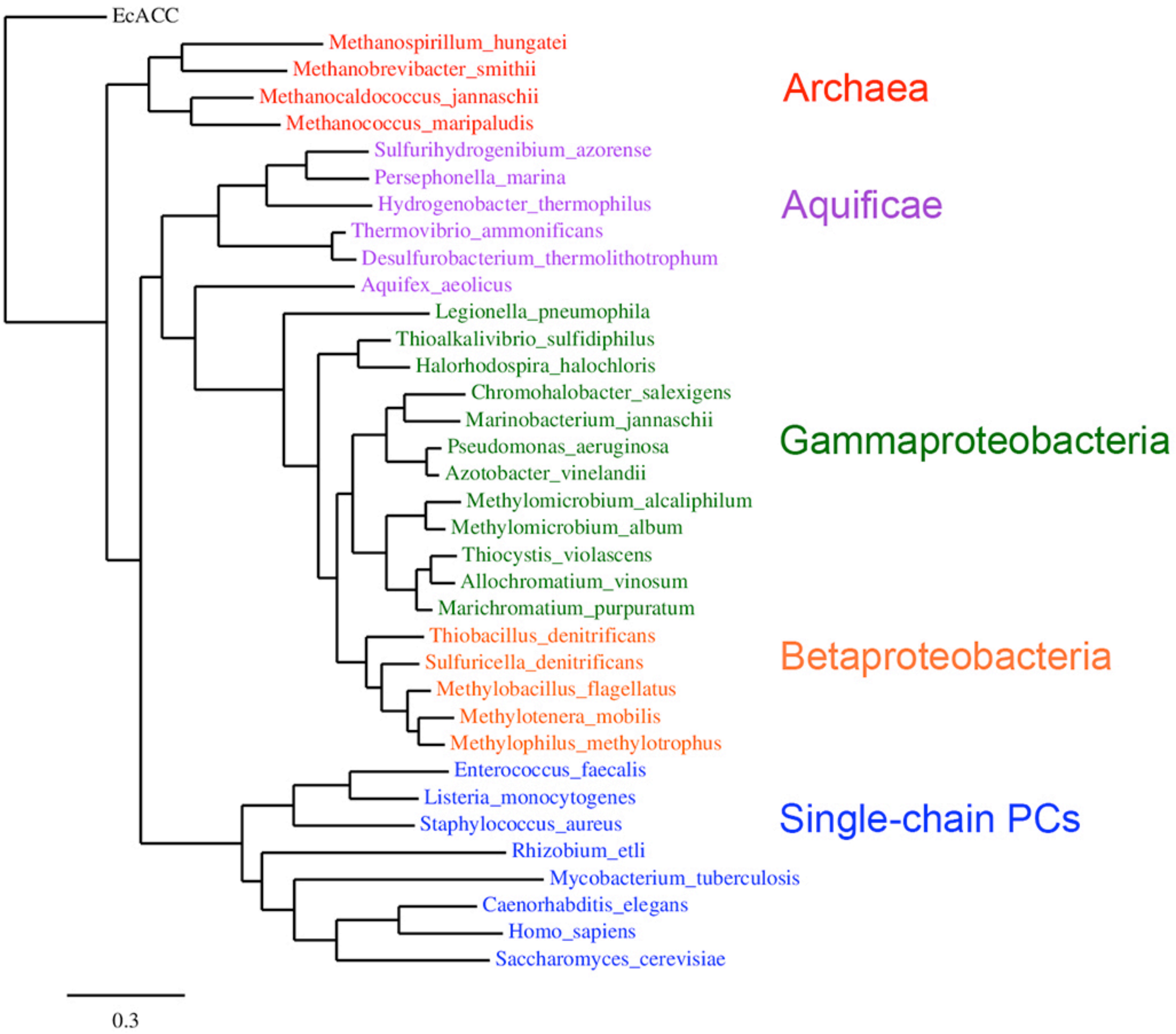

Supplementary Fig. 1. Phylogenetic tree for pyruvate carboxylase enzymes. The phylogenetic tree was constructed for selected bacterial two-subunit and single-chain PC enzymes and archaeal two-subunit PC enzymes based on sequence alignment of the BC domain only. Modified from an output from the Phylogeny.fr server.

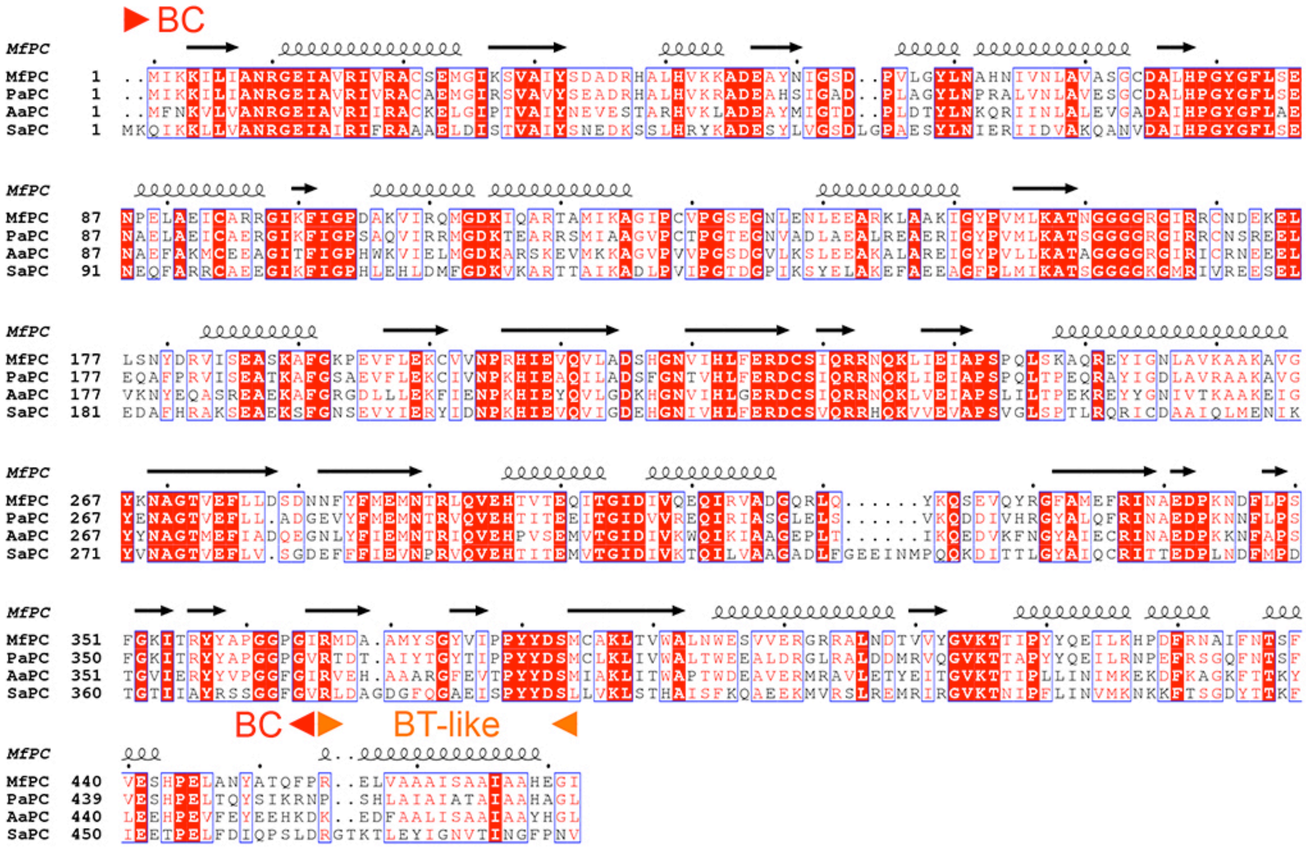

Supplementary Fig. 2. Sequence alignment of the PC α subunit from *Methylobacillus flagellatus* (MfPC), *Pseudomonas aeruginosa* (PaPC), and *Aquifex aeolicus* (AaPC). These species are representative of the classes Betaproteobacteria, Gammaproteobacteria, and Aquificae, respectively. The equivalent region of the single-chain *Staphylococcus aureus* PC (SaPC) is included for comparison. The various domains are labeled. Modified from an output from ESPript [1].

1 Gouet, P., Courcelle, E., Stuart, D. I. & Metoz, F. ESPript: analysis of multiple sequence alignments in PostScript. *Bioinformatics* **15**, 305-308 (1999).

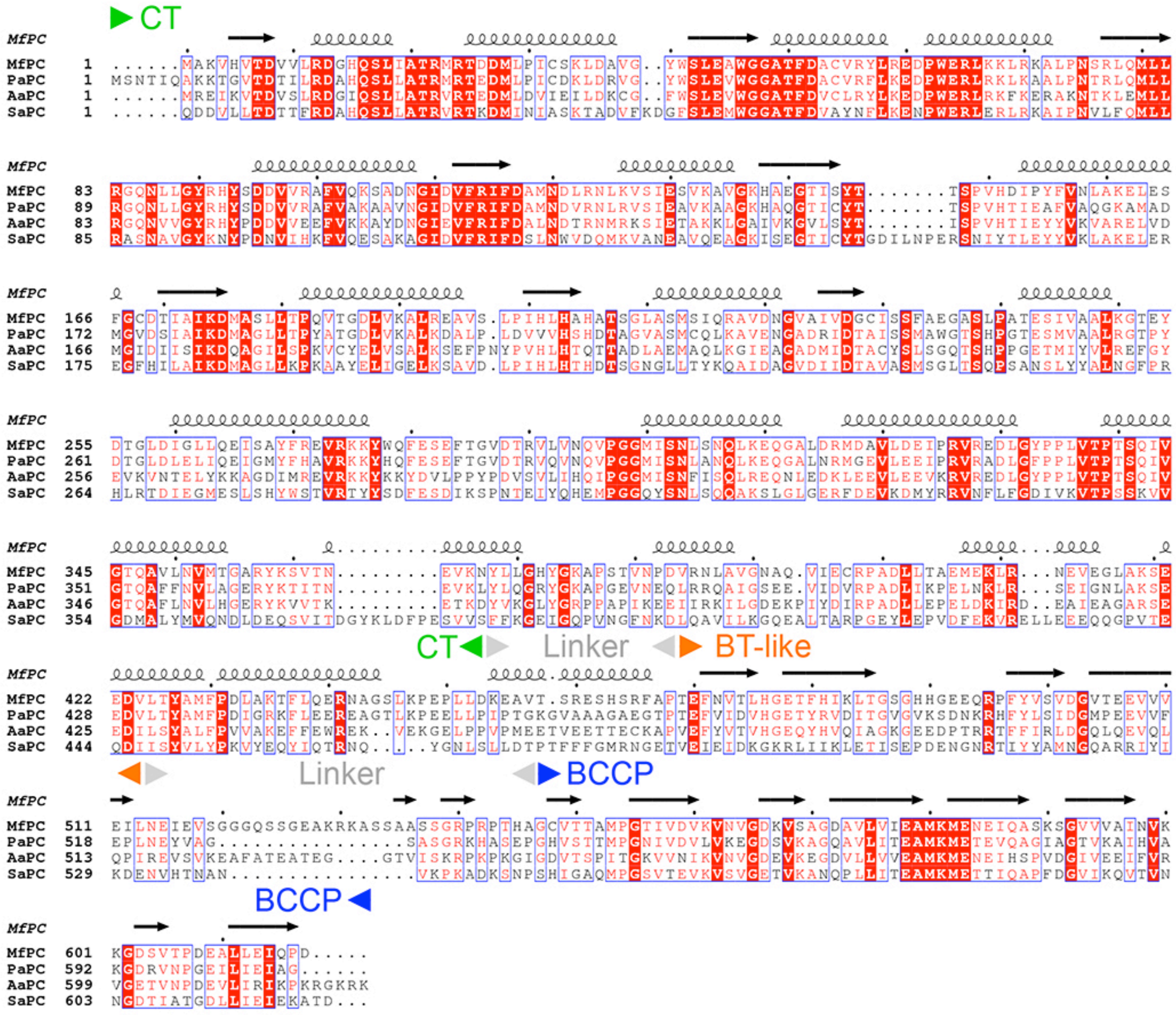

Supplementary Fig. 3. Sequence alignment of the PC β subunit from *Methylobacillus flagellatus* (MfPC), *Pseudomonas aeruginosa* (PaPC), and *Aquifex aeolicus* (AaPC). The equivalent region of the single-chain *Staphylococcus aureus* PC (SaPC) is included for comparison. The various domains are labeled.

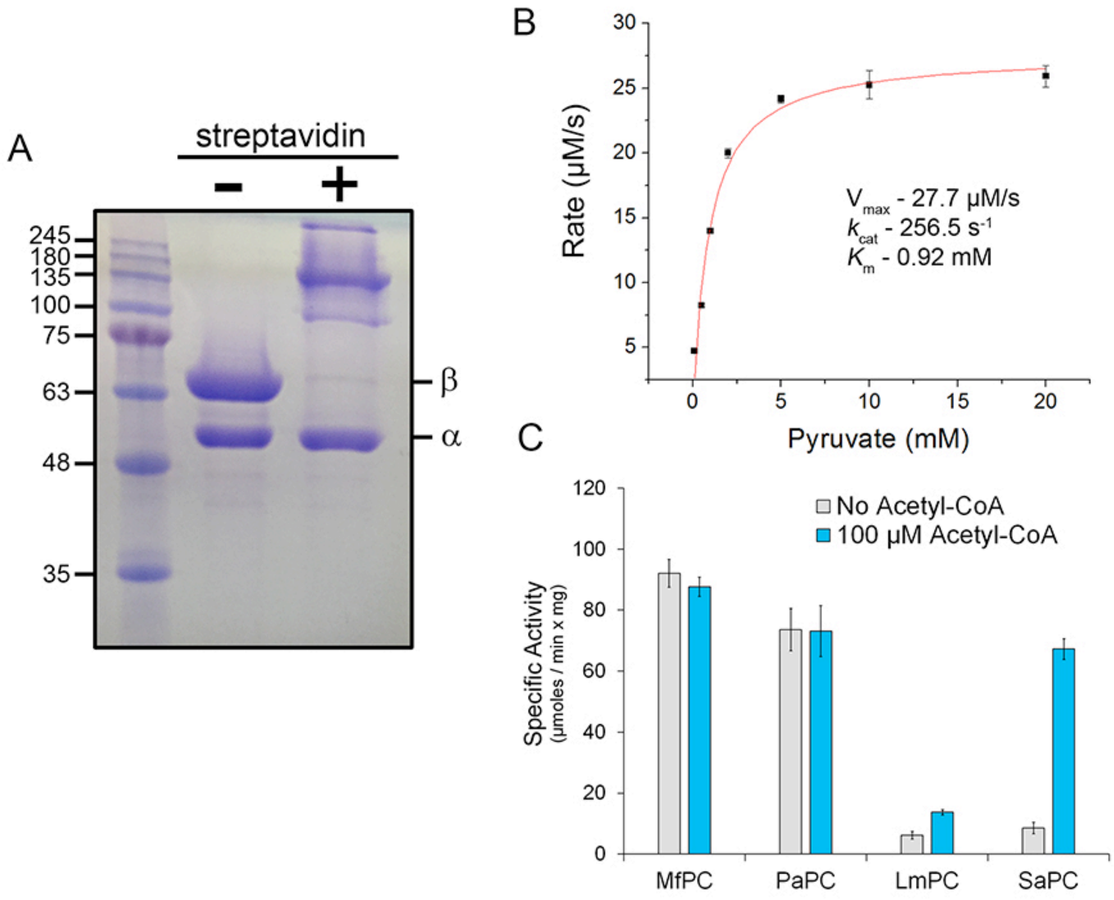

Supplementary Fig. 4. Biochemical characterizations of MfPC. (**a**). Streptavidin gel shift assay. SDS gel showing a shift of the β subunit in the presence of streptavidin, confirming complete biotinylation of the enzyme. (**b**). Michaelis-Menten curve of MfPC. (**c**). Comparing specific activities of MfPC, PaPC, LmPC, and SaPC, either with or without 100 μM acetyl-CoA added to the reaction. The concentration of the pyruvate substrate was at 20 mM.

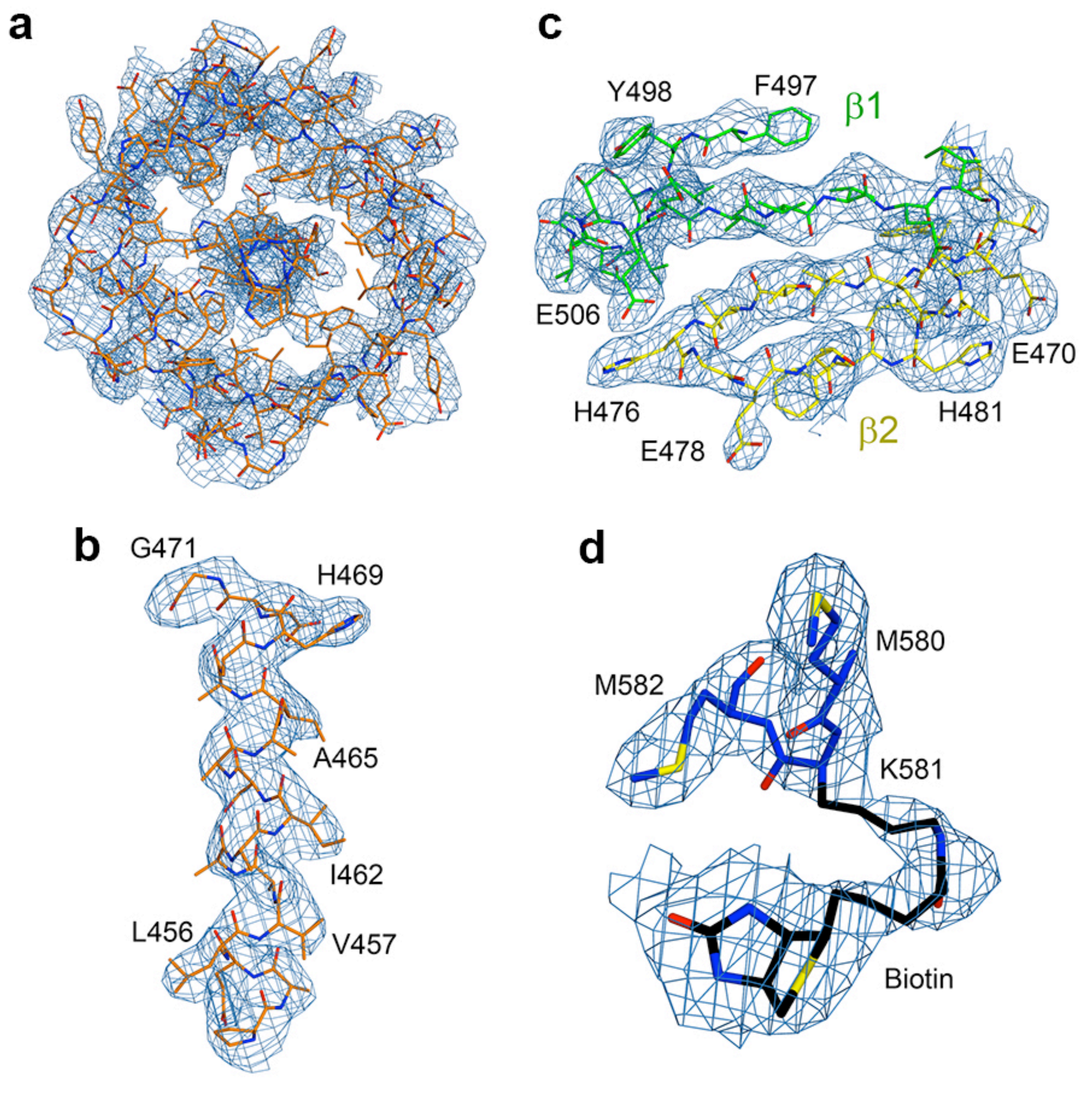

Supplementary Fig. 5. Electron density for selected regions of the mutant MfPC structure. (**a**). $2F_o$–F_c electron density at 3.0 Å resolution for the entire BT-like domain of mutant MfPC, contoured at 1σ. The view is looking down the central helix. (**b**). $2F_o$–F_c electron density for the central helix of the BT-like domain of mutant MfPC. (**c**). $2F_o$–F_c electron density for four β strands of the BT-like domain of mutant MfPC, from two separate β subunits. (**d**). F_o–F_c electron density contoured at 3σ for biotin in the BCCP domain.

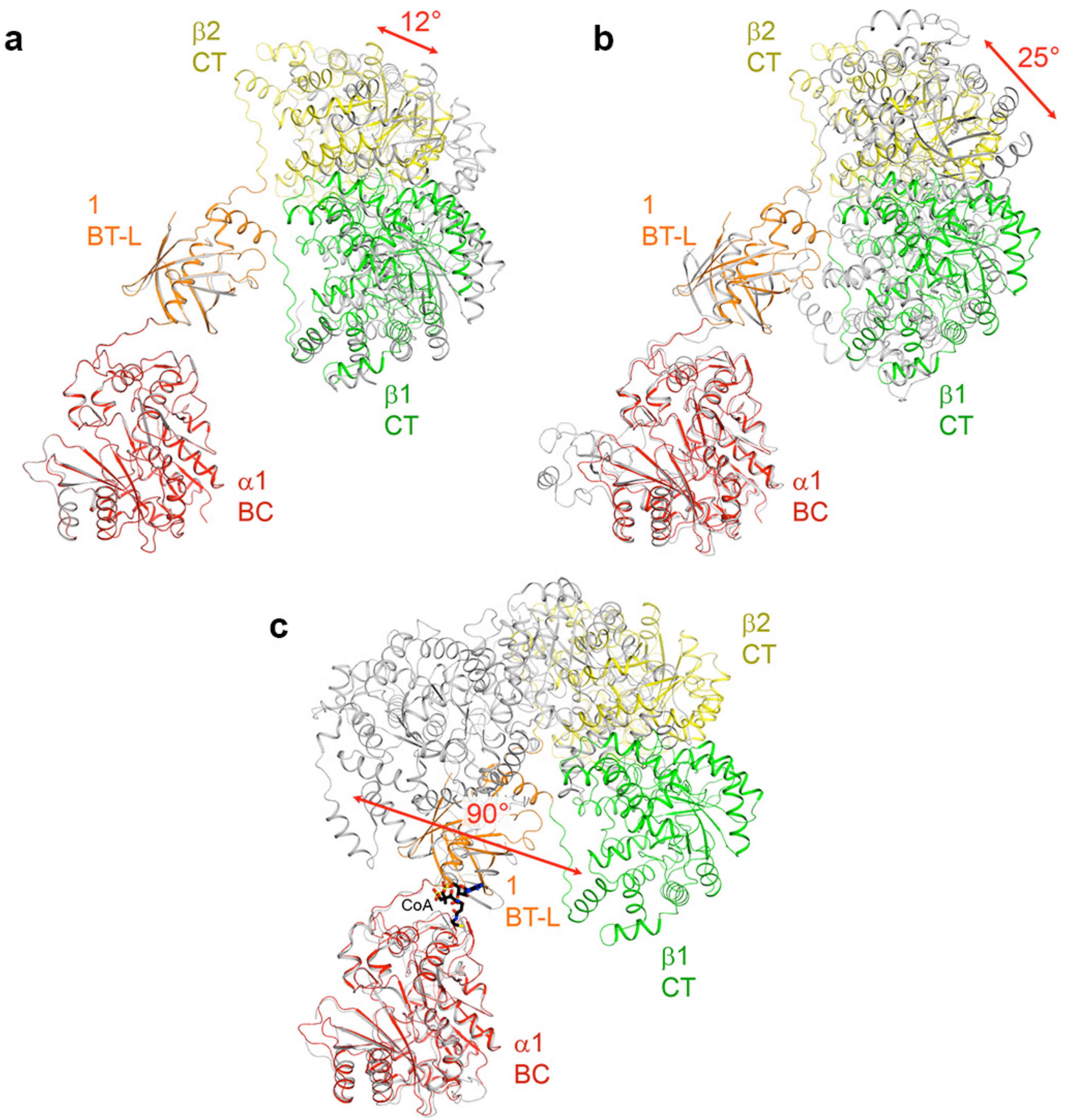

Supplementary Fig. 6. Structural comparisons of the BC-CT dimers of PC. (**a**). Overlay of the two BC monomers of mutant MfPC, showing the difference (red arrow) in the orientation of the two CT dimers. (**b**). Overlay of the BC monomers of mutant (color) and wild-type (gray) MfPC, showing the difference (red arrow) in the orientation of the two CT dimers. (**c**). Overlay of the BC monomers of mutant MfPC (color) and the SaPC-CoA complex (gray), showing the difference (red arrow) in the orientation of the CT dimers. The bound position of the CoA molecule (black) is also shown.

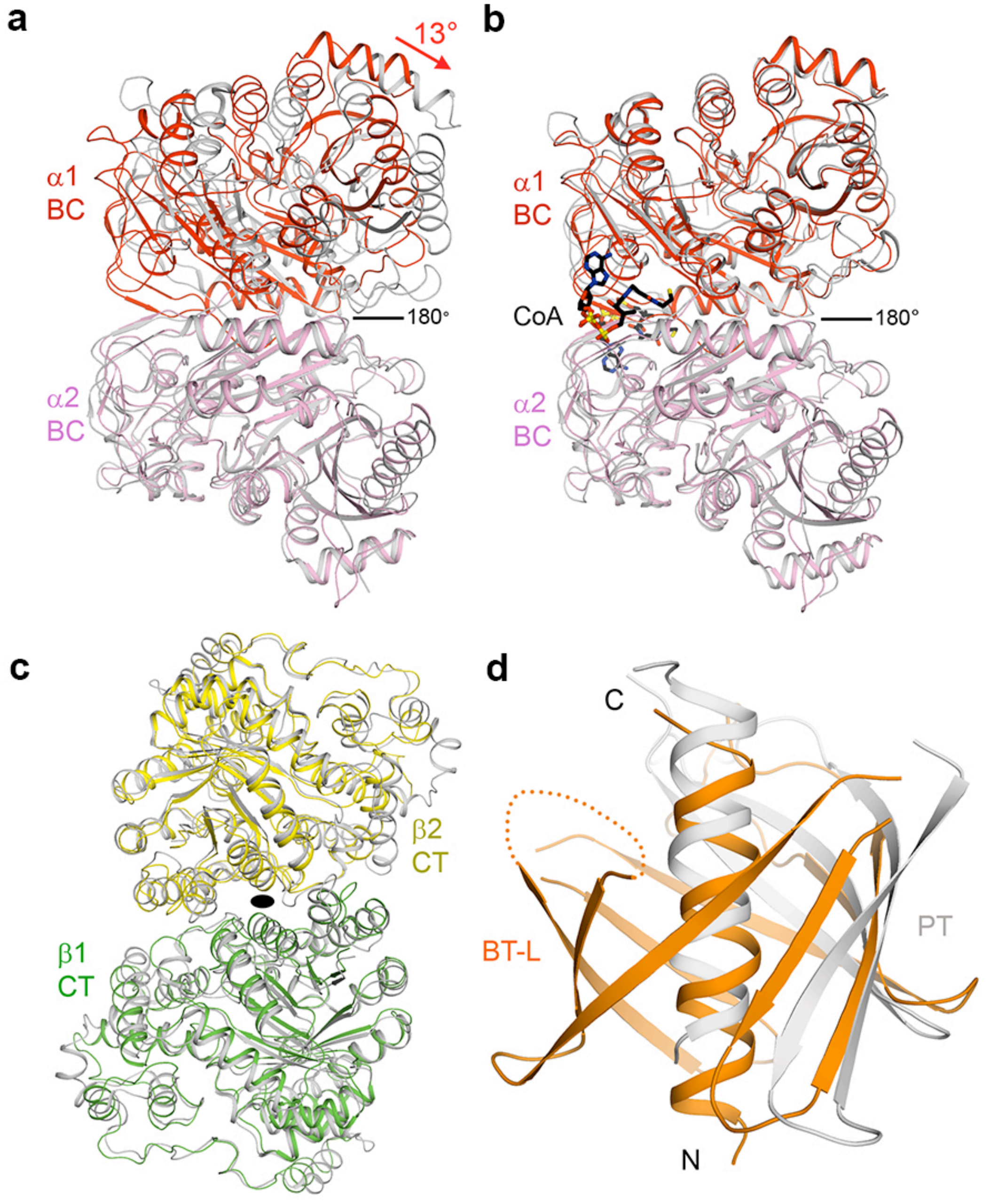

Supplementary Fig. 7. Structural comparisons of domains in MfPC with SaPC. (**a**). Overlay of a BC monomer of MfPC and SaPC (bottom), showing the difference in the position of the second monomer (top). When the entire dimer is overlaid, they have a 1.9 Å r.m.s. distance for equivalent Cα atoms. (**b**). Overlay of a BC monomer of MfPC and SaPC-CoA complex (bottom), showing the difference in the position of the second monomer (top). The bound position of the CoA molecules (black) are also shown. When the entire dimer is overlaid, they have a 1.0 Å r.m.s. distance for equivalent Cα atoms. (**c**). Overlay of the CT dimer of MfPC (colored) and SaPC (gray), with 1.6 Å r.m.s. distance for equivalent Cα atoms. (**d**). Overlay of the MfPC BT-like (BT-L) domain (orange) and the SaPC PT domain (gray), with 3.7 Å r.m.s. distance for 56 equivalent Cα atoms.

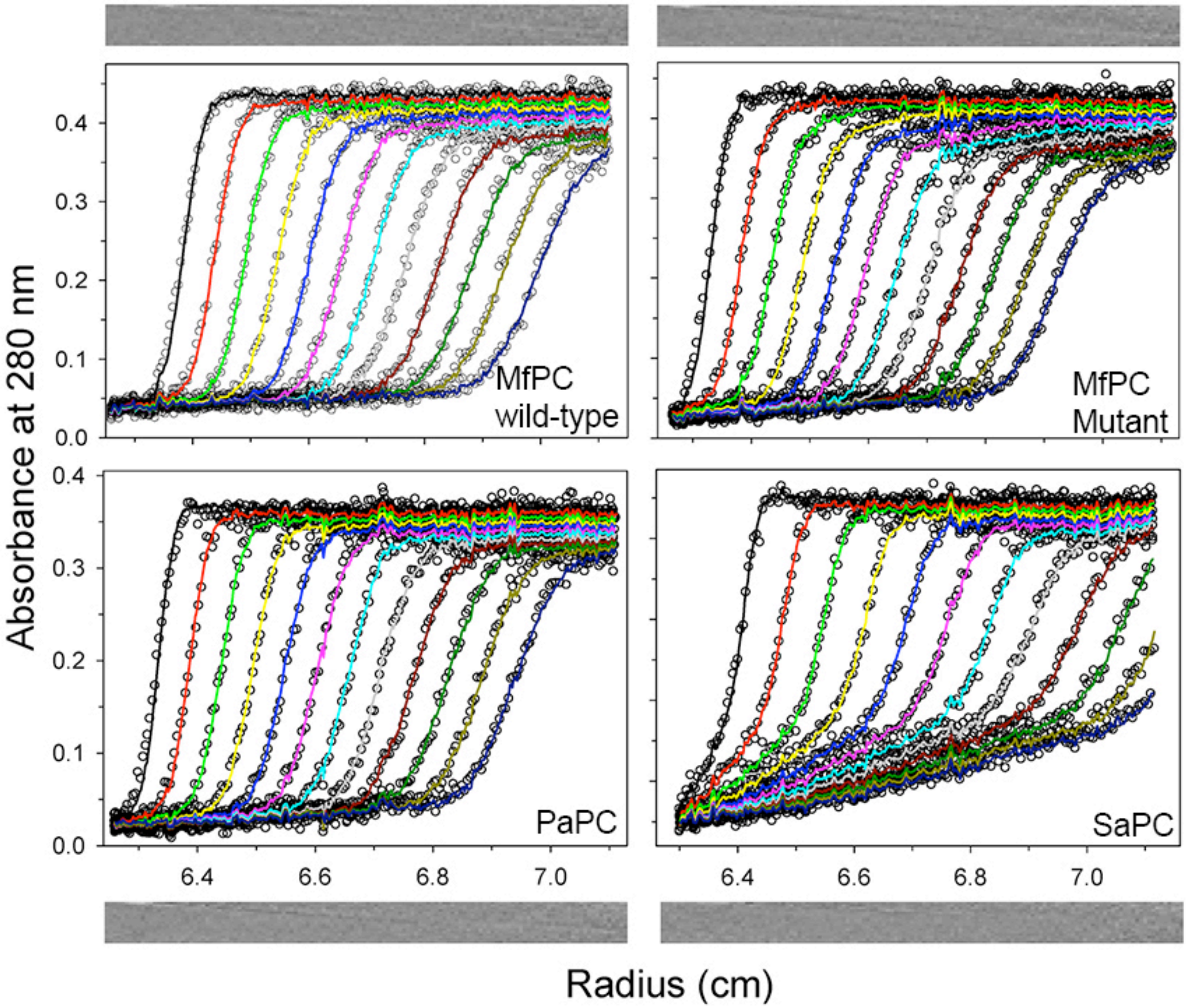

Supplementary Fig. 8. Sedimentation velocity AUC data for MfPC wild-type and mutant, PaPC and SaPC at a concentration of 0.5 mg/ml. The traces of absorbance at 280 nm are shown as open circles and the best-fit to the continous c(s) distribution model are shown as the curves. The gray bar showed the residual bitmap of the raw data and the best-fit results.

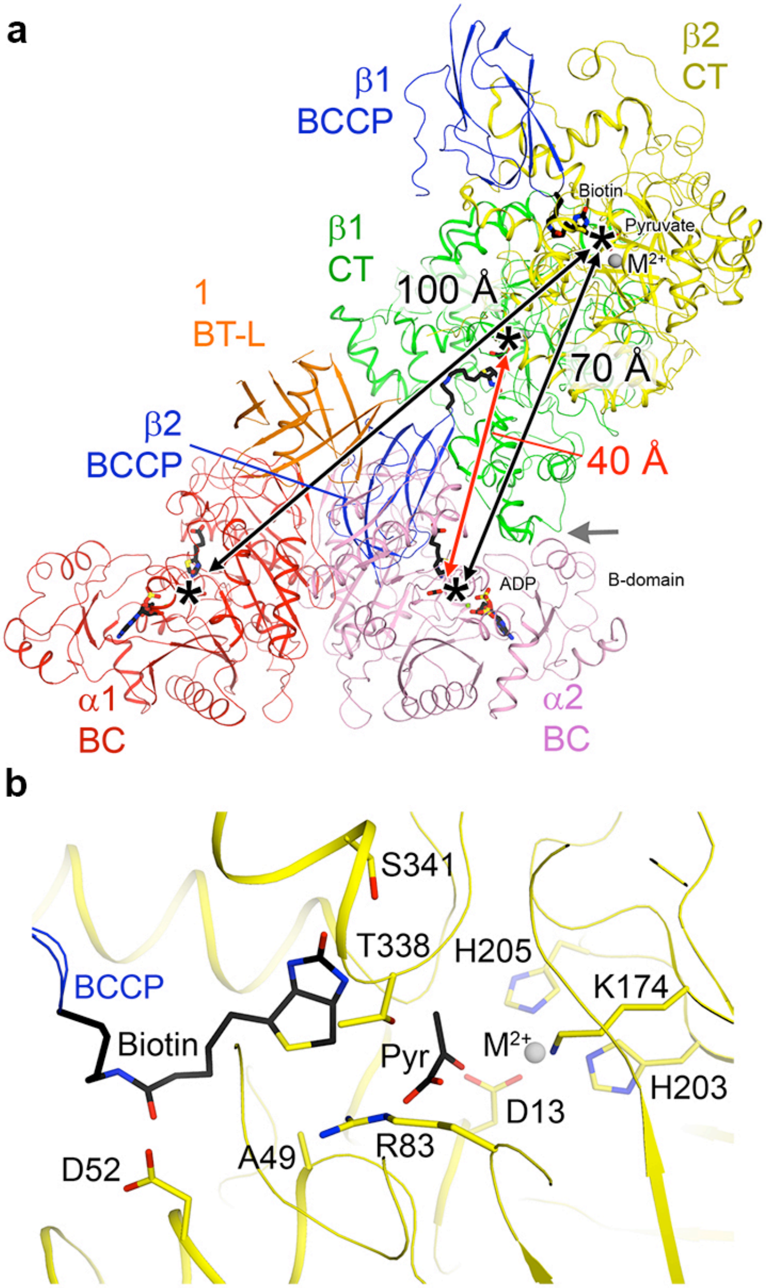

Supplementary Fig. 9. Catalysis by MfPC. (**a**). Movement of BCCP-biotin during catalysis. The BCCP-biotin from the proximal β subunit (β1) is bound to the CT active site of the distal β subunit (β2) in wild-type MfPC structure. A BCCP-biotin (from β2) in the proximal β subunit would clash with the second α subunit (α2). The distances between the BC and CT active sites are indicated with the black and red arrows. The gray arrow indicates contacts between the B domain of BC and the proximal β subunit. The distance between the distal CT active site and the second BC domain (100 Å) is much longer than all the BC-CT active site distances observed so far. (**b**). A close-up view of the β2 CT active site from panel a. Biotin and pyruvate (Pyr) are shown in black, and important residues, including A49, are labeled. The catalytic metal ion (M^{2+}) is shown as a gray sphere.

Supplementary Table 1
Primers used to make pyruvate carboxylase mutants and complementation strains in *Pseudomonas aeruginosa* PA14

Primer	Sequence (5' to 3')	Construct
Δ*PA14_71720-71740* 1F	ccaggcaaattctgttttatcagaccgcttctgcgttctgatGATGATGCCCTTGAACTGCT	In-frame markerless deletion of *PA14_71720-PA14_71740*
Δ*PA14_71720-71740* 1R	gtctactccgaggccgatcAGGCGATGAAGATGGAAACC	
Δ*PA14_71720-71740* 2F	ggtttccatcttcatcgcctGATCGGCCTCGGAGTAGAC	
Δ*PA14_71720-71740* 2R	ggaattgtgagcggataacaatttcacacaggaaacagctCAATTCCTCCACCGGTAGTT	
*PA14_71720-71740*c 1F	ccaggcaaattctgttttatcagaccgcttctgcgttctgatGATGATGCCCTTGAACTGCT	*PA14_71720-PA14_71740* complementation
*PA14_71720-71740*c 1R	cggcgagcgctacaagaccaTCACCAACGAAGTGAAGCTG	
*PA14_71720-71740*c 2F	cagcttcacttcgttggtgaTGGTCTTGTAGCGCTCGCCG	
*PA14_71720-71740*c 2R	tggagttcctgctcgccgacGGCGAGGTGTACTTCATGG	
*PA14_71720-71740*c 3F	ccatgaagtacacctcgccGTCGGCGAGCAGGAACTCCA	
*PA14_71720-71740*c 3R	ggaattgtgagcggataacaatttcacacaggaaacagctCAATTCCTCCACCGGTAGTT	
PA14_71740-K451* 1F	ccaggcaaattctgttttatcagaccgcttctgcgttctgatGACGAAGCCCACAGCATC	K451X point mutation in *PA14_71740* (α subunit)
PA14_71740-K451* 1R	gccaggtgcgacgggttgcgttaGATCGAGTACTGGGTCAGTT	
PA14_71740-K451* 2F	aactgacccagtactcgatctaaCGCAACCCGTCGCACCTGGC	
PA14_71740-K451* 2R	ggaattgtgagcggataacaatttcacacaggaaacagctCCAGGCCATGCTGGAGAT	
PA14_71720-A55T 1F	ccaggcaaattctgttttatcagaccgcttctgcgttctgatCTCGAGAAGTGCATCGTCAA	A55T point mutation in *PA14_71720* (β subunit)
PA14_71720-A55T 1R	cgcacgcaggcgtcgaaggtagtGCCGCCCCAGACTTCCAGCG	
PA14_71720-A55T 2F	cgctggaagtctggggcggcactACCTTCGACGCCTGCGTGCG	
PA14_71720-A55T 2R	ggaattgtgagcggataacaatttcacacaggaaacagctCGCACGTCGATGACTTCCT	
PA14_71720-K572A 1F	ccaggcaaattctgttttatcagaccgcttctgcgttctgatCTGAAGGCCGTGGAAAAC	K572A point mutation in *PA14_71720* (β subunit)
PA14_71720-K572A 1R	gcctggacctcggtttccatggcCATCGCCTCGGTGATCAACA	
PA14_71720-K572A 2F	tgttgatcaccgaggcgatggccATGGAAACCGAGGTCCAGGC	
PA14_71720-K572A 2R	ggaattgtgagcggataacaatttcacacaggaaacagctATGATGCCCTTGAACTGCTC	
